# With a little help from my friends: cooperation can accelerate the rate of adaptive valley crossing

**DOI:** 10.1186/s12862-017-0983-2

**Published:** 2017-06-17

**Authors:** Uri Obolski, Ohad Lewin-Epstein, Eran Even-Tov, Yoav Ram, Lilach Hadany

**Affiliations:** 1 Department of Molecular Biology and Ecology of Plants, Tel-Aviv University, 6997801 Tel Aviv, Israel; 2 Current address: Department of Zoology, University of Oxford, Oxford, UK; 3 Department of Molecular Microbiology and Biotechnology, Tel-Aviv University, Tel-Aviv, Israel; 4 Present Address: Department of Biology, Stanford University, Stanford, CA USA

**Keywords:** Peak shift, Adaptive landscape, Cooperation, Rugged fitness landscape, Mathematical models/simulations, Altruism

## Abstract

**Background:**

Natural selection favors changes that lead to genotypes possessing high fitness. A conflict arises when several mutations are required for adaptation, but each mutation is separately deleterious. The process of a population evolving from a genotype encoding for a local fitness maximum to a higher fitness genotype is termed an *adaptive peak shift*.

**Results:**

Here we suggest cooperative behavior as a factor that can facilitate adaptive peak shifts. We model cooperation in a *public goods* scenario, wherein each individual contributes resources that are later equally redistributed among all cooperating individuals. We use mathematical modeling and stochastic simulations to study the effect of cooperation on peak shifts in both panmictic and structured populations. Our results show that cooperation can substantially affect the rate of complex adaptation. Furthermore, we show that cooperation increases the population diversity throughout the peak shift process, thus increasing the robustness of the population to sudden environmental changes.

**Conclusions:**

We provide a new explanation to adaptive valley crossing in natural populations and suggest that the long term evolution of a species depends on its social behavior.

**Electronic supplementary material:**

The online version of this article (doi:10.1186/s12862-017-0983-2) contains supplementary material, which is available to authorized users.

## Background

Adaptive landscapes, introduced by Sewall Wright in the 1930s [1], are a useful metaphor for the relationship between genotype and fitness. Under this analogy, fitness is portrayed as a function of genotype, varying between different allele combinations. Complex traits — which depend on two or more loci — can produce rugged adaptive landscapes due to fitness interactions between loci. The simplest instance of a rugged fitness landscape consists of two loci in which mutations are jointly beneficial but separately deleterious. Adaptive valley crossing, also known as *adaptive peak shift*, has long been an evolutionary conundrum: how can a population evolve to a higher fitness optimum if it has to “cross” a less fit genotype on the way?

Two main stages are needed for such an evolutionary process [2]. First, the fitter genotype must appear in the population. This could happen as a result of sequential mutations in the same lineage, by recombinant offspring of mutant parents, or by migration from another population. Second, the fitter genotype will have to spread in the population. However, these two stages have opposing optimal conditions [3]. Mild selection facilitates the first stage of the process: single mutants are more likely to survive in a mild selection regime, increasing the rate of appearance of the fitter genotype by recombination or by acquisition of an additional mutation. On the contrary, the second stage is impeded when selection is mild because the fixation probability of a rare genotype decreases [4].

In contrast to Wright, some researchers have suggested that adaptive landscapes, which can be extended to various topologies in multidimensional genotype spaces [5–8], might most commonly be single peaked [9–12]. However, these theories cannot account for all adaptive landscape topologies, and do not exempt evolutionary biologists from a characterization of evolution on a given rugged adaptive landscape.

Wright himself was the first to offer a theoretical solution to the adaptive peak shift problem [1]. His solution, the *Shifting Balance Theory*, was based on a subdivision of the population into small demes in which mutation and random genetic drift can bring the population to the base of the “peak”. Then, mutation and natural selection allow the deme population to climb the higher “peak”, and migration can allow the new genotype to spread to other demes. While the *Shifting Balance Theory* has been demonstrated to work in specific cases [13–15], the range of realistic peak shift scenarios it explains is controversial [16, 17].

Additionally, considerable amounts of research has focused on finding unique conditions which can facilitate adaptive valley crossing: Dividing the population into smaller subpopulations connected by migration was shown to increase the rate of adaptive valley crossing, even without Wright’s assumptions of increase and decrease in deme sizes as a function of the beneficial genotypes inhabiting them [18]; Furthermore, dividing the population into only two populations connected by migration, but changing the selection pressure on each population, was also found to substantially reduce the waiting time for a peak shift [19]; Mutation or recombination rates that increase with low fitness were shown to facilitate peak shifts, as this entails that less fit individuals can more rapidly adapt and traverse the fitness valley [20, 21]; Finally, assortative mating was also found to increase the rate of adaptive peak shifts in diploid populations, as it increases the mating between individuals of the advantageous genotype, thus preventing recombination from breaking advantageous allele combinations [22]. Further theoretical research by Weissman et al. has elaborated the various possible peak shift dynamics, and established the rate of adaptive valley crossing for sexual [23] and asexual [24] populations under different ranges of evolutionary forces such as selection, mutation, recombination and population size.

Crossing adaptive valleys can occur through two types of dynamics, driven by either drift (when selection is weak) or selection (when selection is strong enough). In the first case, the single mutants can increase to a relatively high frequency by drift, from which the double mutants can emerge by either mutations or recombination between single mutants, and eventually take over [23, 24]. In contrast, when selection is strong, the single mutants are found at low frequencies. Hence, double mutants rarely appear, and when they appear they might often be decomposed by recombination when a double mutant and a wild-type mate [25, 26]. When selection is stronger than recombination, the double mutant can take over the population without the single mutants reaching high frequencies (also referred to as “*stochastic tunneling*” [27, 28]), and this process might be orders of magnitude faster than drift-driven peak shift [23]. In this study we focus on the latter dynamics.

We consider an additional factor that can affect the process of crossing adaptive valleys: cooperative behavior. We show that for a population subdivided into demes and connected by migration, cooperation between individuals within the same deme can considerably increase the rate of adaptive peak shifts. Note that we assume cooperation has either already evolved and fixated, or will not evolve, in the populations in question. We do not model the evolution and spread of cooperative behavior.

We focus on a *public goods* form of cooperation [29]: all individuals within a deme contribute some resources (thus contributing fitness) to other deme members, and all deme members receive an equal amount of the redistributed resources. This cooperative behavior reduces the fitness difference between different genotypes and therefore effectively “smooths” the landscape to some degree. As a result, less fit mutants are more likely to survive with cooperation, eventually increasing the rate of appearance of the fittest genotype.

Cooperation has an opposite effect on the fixation of the fittest genotype. Precisely because cooperation smooths the adaptive landscape, it reduces the relative advantage of the fittest genotype, and with it, its fixation probability. Nevertheless, we show that for intermediate levels of cooperation the increase in the rate of appearance of the fittest genotype outweighs the decrease in its fixation probability, and altogether shortens the total adaptation time. Furthermore, we find that smoothing the adaptive landscape serves the cooperative population in another sense: it increases the population diversity. This increase in diversity is beneficial in evolutionary terms, as it can help populations to overcome environmental changes, parasites, etc. [30].

Overall, our results show that cooperation affects adaptive peak shifts substantially and might be an important and overlooked component of complex adaptation.

## Methods

We model a population of sexually reproducing haploid individuals, containing two bi-allelic loci. Before considering cooperation, the basic fitness of the wild-type *ab* is 1; the fitness of the single mutant genotypes, *Ab* and *aB*, is 1 − *s*; and the fitness of the double mutant *AB* is 1 + *sH*. *s* and *H* are the selection coefficient of the single mutant and the double mutant coefficient, respectively (*s* > 0 , *H* > 0). We assume equal forward and backward mutation rates for both loci (defined in units of mutations per generation per locus), and denote them by *μ*. Recombination occurs with rate *r* per generation per loci pair.

We model a population composed of *n* demes connected by migration, each of size *k*. Thus, the total size of the population is *N* = *n* · *k*. Each generation, individuals of the same deme cooperate. We model cooperation using a *public goods* scenario [29]: Each individual in the deme contributes a constant fraction *c* of its baseline fitness to the deme (0 ≤ *c* ≤ 1), and this fraction is hereafter referred to as the ‘cooperation level’; the sum of all contributions is multiplied by a constant *b* (*b* ≥ 1); finally, this sum is equally redistributed between the deme members. Hence, *b* determines the fold-increase of contributed resources due to cooperation.

In our model, the fitness of each individual is determined by its genotype and the effect of cooperation. We denote *ω*_*i*_, as the original fitness of individual *i*, determined by its genotype (*ab* , *Ab* , *aB* , *AB*), and derive the fitness *ω*_*i* , *d*_ of individual *i* in deme *d*, after considering cooperation:

1 $$ {\omega}_{i, d}={\omega}_i\cdotp \left(1- c\right)+\frac{b}{k}\sum_{j\in d}{\omega}_j\cdotp c $$

After cooperating and undergoing selection, individuals migrate to other demes with probability *m* and mate. Setting *m* = 1 − 1 ⁄ *n* describes a panmictic population, in which offspring are randomly regrouped to new demes every generation and mate. For simplicity, we assume that migration to each deme is equiprobable. The offspring generation replaces the parent generation, so that population size remains constant and generations do not overlap (for parameter description s﻿ee Table 1).

**Table 1:** Parameters used in the analytical model and the stochastic simulation

Parameter	Description
*n*	Number of demes
*k*	Size of each deme
*μ*	Mutation rate
*r*	Recombination rate
*s*	Selection coefficient
*H*	Double mutant coefficient
*c*	Fraction of resources contributed - the level of cooperation
*b*	Contributed resources multiplier - the cooperation benefit
*m*	Migration rate

In our model we focus on parameters for which the peak shift is mainly driven by selection (for detailed assumptions and analysis see Additional file 1: SI1a). Since in this scenario the single mutants are found at low frequencies in the population, double mutants emerge at a low rate. Within this framework, we first analyzed the expected waiting time for a peak shift in a panmictic population with demes of size *k*. We did so by estimating the expected waiting time for the appearance of a first double mutant, and its fixation probability, similarly to the method presented in [19]. Using these two estimates, we approximated the total adaptation time.

We next investigated the peak shift process in populations with limited migration rates, which lead to inter-deme diversity [31, 32], rendering our analytic approximations no longer compatible. We therefore approached this analysis by developing a Wright-Fisher stochastic simulation, which considers natural selection, migration, mating, recombination, mutation and drift (see Additional file 1: SI2). The simulation comprises three stages. In the first stage a population inhabited by wild-types evolves towards a mutation-selection balance. In the second stage, we simulate the population until a double mutant appears for the first time. This allows us to estimate the expected time for the appearance of a double mutant. In the third stage, the double mutant either becomes extinct or fixates in the population (determined by reaching a frequency of 0.99). From this stage we can estimate the probability that a double mutant will fixate (see Additional file 1: SI2). Combining the two measures (expected first appearance time and fixation probability) we can estimate the expected waiting time for the appearance of a double mutant that fixates [19]. We compared simulation results to the analytical approximation for a panmictic population (*m* = 1 − 1/*n*) and they were in close agreement (Additional file 1: SI1b).

We denote the adaptation time in a population with cooperation level *c* by *τ*_*c*_. The relative difference between the adaptation time of a cooperative population with cooperation level *c* > 0 (*τ*_*c*_) and a non-cooperative population (*τ*_0_) is denoted by $\rho (c):=\frac{\tau_0-{\tau}_c}{\tau_0}$.

## Results

We explore the effect of cooperation on the rate of peak shifts, in the presence of strong selection (see Additional file 1: SI1a). First, we analyze a panmictic population scenario, where the demes are completely mixed by migration every generation, to which we produce an analytical approximation and interpret the influence of various parameters on the results. In the second part of the results we use stochastic simulations to examine the effect of cooperation on peak shift dynamics of demes connected by limited migration, and on the level of diversity during the process.

### A panmictic population

We start with the special case where the population is panmictic, i.e. after individuals cooperate in demes of size *k*, they disperse and randomly regroup into new demes, where they mate. When the selective disadvantage of the single mutant (*s*) decreases, more single mutants are found in the population, and the expected waiting time for the appearance of a double mutant decreases. Analogously, as the selective advantage of the double mutant (*sH*) increases, so does its fixation probability (see Additional file 1: SI1a for details).

Differently from the model presented in [19], here the fitness of each individual is not entirely determined by its genotype, but also depends on the composition of deme members in the deme it inhabits, through cooperation in a “public goods” scenario (as expressed in Eq. 1). Therefore, the fitness of a genotype cannot be described by a single value. We thus use the mean fitness of each genotype, averaging over all possible deme compositions. We define $\overset{\sim }{s}$ and $\overset{\sim }{sH}$ as the effective selection coefficients, i.e. the average disadvantage of the single mutants and the average advantage of the double mutants, relative to the wild-type, after cooperation has taken place (See Additional file 1: SI1a for formal derivation). We present an approximation for $\overset{\sim }{s}$ as a function of the original selection coefficient (*s*) and the cooperation (*c*, *b*, *k*) at mutation-selection balance (before double mutants first appear or when they are still rare) (Additional file 1: SI1a.1):

2 $$ \overset{\sim }{s}\approx s\cdotp \frac{1- c+\frac{cb}{k}}{1- c+ cb} $$

Our analysis also revealed that $\overset{\sim }{s H}=\overset{\sim }{s} H$ (i.e. the effective selection coefficient of the double mutant is proportional to that of the single mutant). Therefore, any changes induced by cooperation on the single mutant disadvantage effect the double mutant advantage linearly (Additional file 1: SI1a.1). Equation 2 demonstrates that increasing the cooperation level (*c*) decreases both effective selection coefficients.

Hence, cooperation has contrasting effects over the two main stages of the peak shift: First, it reduces the disadvantage of the single mutants, therefore increasing their survival. As a result, the waiting time for the first double mutant shortens in comparison to a non-cooperative population (Additional file 1: SI1a.2). On the other hand, cooperation reduces the benefit of the double mutant, thus decreasing its fixation probability (Additional file 1: SI1a.3).

**Fig. 1:**
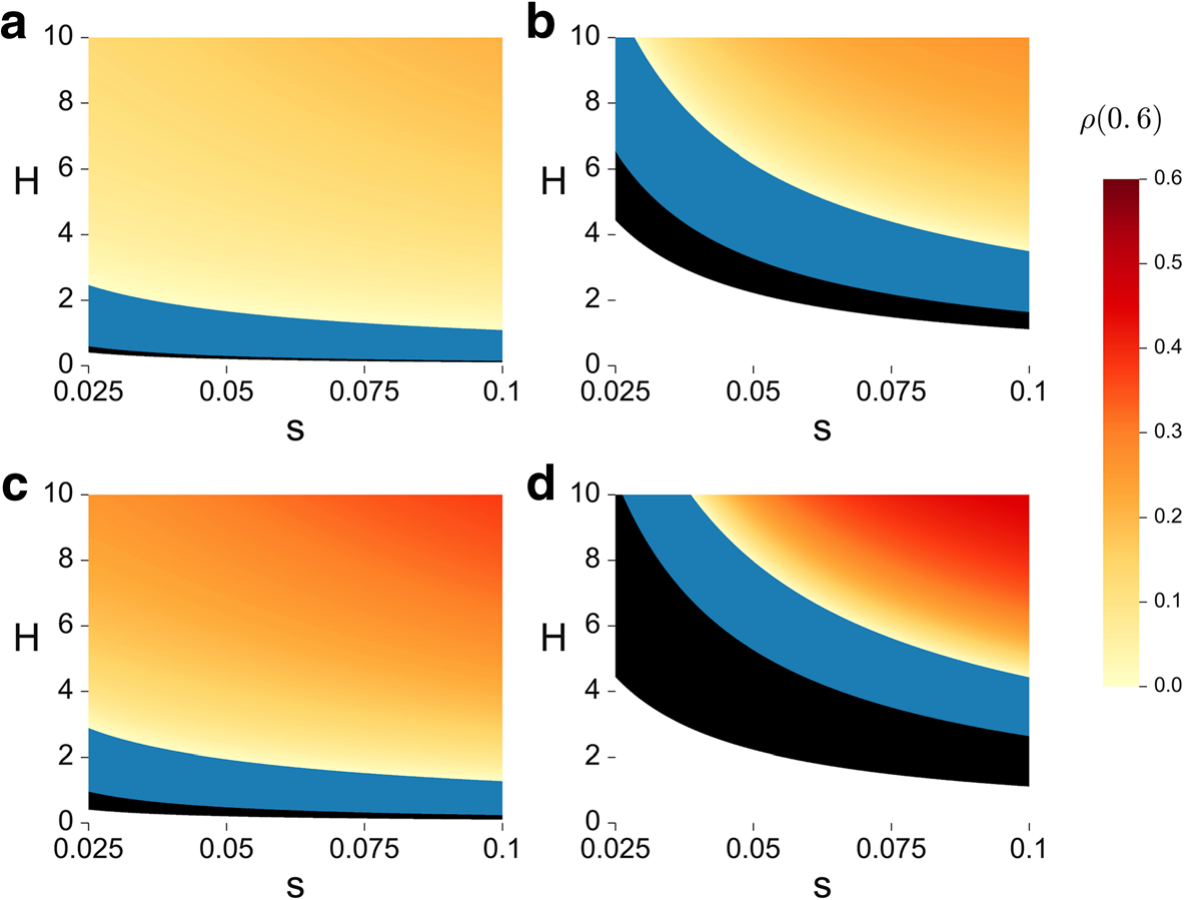
Cooperation and selection affect adaptation time in panmictic populations. The relative difference in adaptation time due to cooperation (*ρ*(0.6)) is plotted (color coding) as function of the selection coefficient, *s* (x-axis), and the double mutant coefficient, *H* (y-axis). Results are based on analytical approximations (Additional file 1: SI1a) of a panmictic population. White areas represent parameters where both cooperators and non-cooperators are not expected to achieve a peak shift by the process described in the text; black areas are parameters for which only non-cooperators are expected to go through the peak shift process; blue areas represent parameters for which both cooperators and non-cooperators achieve a peak shift, but non-cooperators accomplish the process faster. Yellow to orange areas are parameters for which cooperators achieve peak shift faster than non-cooperators, and the colors represent the relative difference in the expected time to peak shift due to cooperation (*ρ*). Results are shown for two recombination rates: *r* = 0.01 (panels **a** and **c**) and *r* = 0.1 (panels **b** and **d**); and two deme sizes: *k* = 2 (panels **a** and **b**) and *k* = 10 (panels **c** and **d**). Color results (yellow-orange and blue) are based on our approximation Additional file 1: SI1a; White and black border are based on SA3 and S28. Additional parameters are: *n* = 10,000/*k* , *μ* = 10^−5^ , *c* = 0.6 , *b* = 1.2

The relative difference in adaptation time due to cooperation, *ρ*(*c*), can be estimated using Eqs. 1 and 2 (see Additional file 1: SI1a.4). Fig. 1 presents *ρ*(*c* = 0.6), derived from our approximation, as a function of the selection coefficient (*s*), and the double mutant coefficient (*H*), for two recombination rates and two deme sizes. When selection against the single mutant and the double mutant coefficient are high (so that *sH* is high, upper right corners in Fig. 1), the decrease in waiting time for the double mutant’s first appearance is more pronounced than the decrease in its fixation probability, resulting in faster adaptation in cooperative populations and thus a higher *ρ* value. When selection is too weak, we find conditions allowing for a peak shift only in a non-cooperative population. In this case a cooperative population is not expected to cross the adaptive valley through the peak shift process described in our model, but rather through a drift-driven process [19, 23] (Fig. 1, black areas, based on eq. S28 in Additional file 1: SI1a and [19]).

The deme size (*k*) affects the peak shift process by changing the influence of rare individuals on the fitness gain from cooperation. In cooperative populations, increasing deme size reduces both the disadvantage of single mutants and the advantage of double mutants (that are rare in the critical stage of the peak shift, when they first appear). Differently, varying deme size has no influence on the effective selection coefficients in a non-cooperative population (as can be seen in eq. 2, when *c* = 0).

The effect of deme size on *ρ* depends on selection. When selection is strong (high *s* , *H* values), larger deme sizes decrease the waiting time for the first appearance of a double mutant significantly, while still retaining sufficient fitness advantage for the double mutant to fixate. Thus we see that under strong selection, increasing the deme size increases *ρ*(*c*) (compare yellow-orange areas in Fig. 1a to c and Fig. 1b to d; see also Additional file 1: Figure S7 for additional results with *k* = 5 , 100). Increased deme size when selection is mild, on the other hand, might decrease the double mutants’ advantage to a point where it is too low to allow a selection-driven peak shift. Thus, even though large demes can hasten the peak shift process of a cooperative population when selection is strong, they can also preclude selection-driven peak shifts when selection is mild (compare black areas in Fig. 1a to c and Fig. 1b to d; see also Additional file 1: Figure S7 for additional results with *k* = 5 , 100).

Recombination can affect the peak shift process in different directions. It has been previously established that intermediate recombination rates can accelerate the peak shift process, relative to very low recombination rates [23]. However, when the recombination rate – acting to break the beneficial mutation combination – exceeds the selective advantage of the double mutant, it can dramatically reduce the fixation probability of a rare double mutant [19, 23, 25]. In that case drift would be the major mechanism for peak shift, and adaptation time might be very long [19, 23]. In Fig. 1 we can see that the parameter ranges of *s* and *H* where both populations do not reach a peak shift under the process we model, expand with higher recombination (Fig. 1, white areas – compare a to b and c to d). Moreover, the selective advantage of the double mutant is further diminished in a cooperative population (Eq. 2). Thus cooperation restricts the parameter range where a population can attain a peak shift through the process we describe (Additional file 1: SI1a), as exhibited by the black areas in Fig. 1. Finally, we observe that the parameter range under which cooperation accelerates the peak shift process, relative to a non-cooperative population, is widened for lower recombination rates (compare yellow regions of Fig.1a, c to b, d).

### A population with limited migration

In this section we expand our investigation of the influence of cooperation on adaptation rates to include populations divided into demes with limited migration between them. Limited migration leads to inter-deme diversity [31, 32], and hence we use stochastic simulations to study such populations (see Methods).

**Fig. 2:**
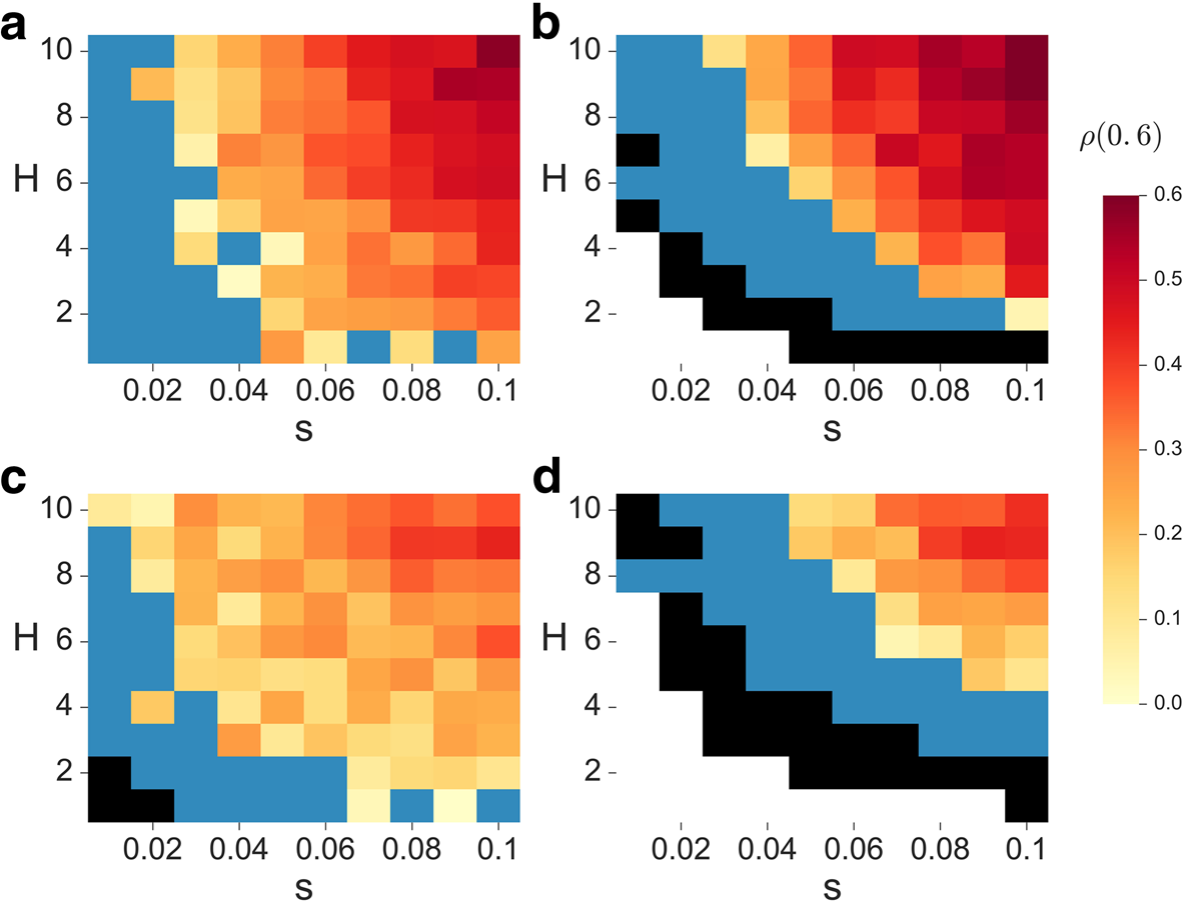
Cooperation and selection affect adaptation time in populations with limited migration. The relative difference in adaptation time due to cooperation (*ρ*(0.6)) is plotted (color coding) as function of the selection coefficient, *s* (x-axis), and the double mutant coefficient, *H* (y-axis). Results are shown for populations with demes of size *k* = 10, and limited migration. Left and right columns correspond to low and high recombination rates (*r* = 0.01, panels **a** and **c**; *r* = 0.1, panels **b** and **d**), whereas the top and bottom rows correspond to low and intermediate migration rates (*m* = 0.01, panels **a** and **b**; *m* = 0.25, panels **c** and **d**). *White* areas represent parameters where both cooperators and non-cooperators are not expected to achieve a peak shift; *black* areas are parameters for which only non-cooperators are expected to shift a peak; *blue* areas represent parameters for which both cooperators and non-cooperators achieve a peak shift, but non-cooperators do so faster. *Yellow* to *red* areas are parameters for which cooperators achieve a peak shift faster than non-cooperators, and the color represents the average relative difference in the expected time for a peak shift due to cooperation, *ρ*(0.6). The results are based on stochastic simulations (Additional file 1: SI2), averaged over ≥ 1200 simulations per parameter set. Additional parameter values are *μ* = 10^−5^ , *b* = 1.2 *c* = 0.6 , *n* = 1000

First, we examine the effects of selection (*s* and *H*) on the adaptation time with and without cooperation. Figure 2 shows the relative difference in adaptation time due to cooperation, *ρ*, for populations with low and intermediate migration rates. Similarly to Fig. 1, *ρ* is a function of the selection coefficient, *s*, on the horizontal axis, and the double mutant coefficient, *H*, on the vertical axis. Limited migration retains the properties exemplified for a panmictic population; high recombination values narrow the range of conditions leading to a selection-driven peak shift (Fig. 2 compare a to b and c to d), and cooperation cannot extend the parameter range enabling the population to cross the adaptive valley under the process described here (Fig. 2, black areas). We can see that where cooperation accelerates complex adaptation relative to non-cooperative populations, the effect is stronger with limited migration; i.e., populations with lower *m* attain higher *ρ* values (note the red-yellow hues of Fig. 2a and b in comparison to Fig. 2c and d; compare Fig. 2 to Fig. 1c and d).

**Fig. 3:**
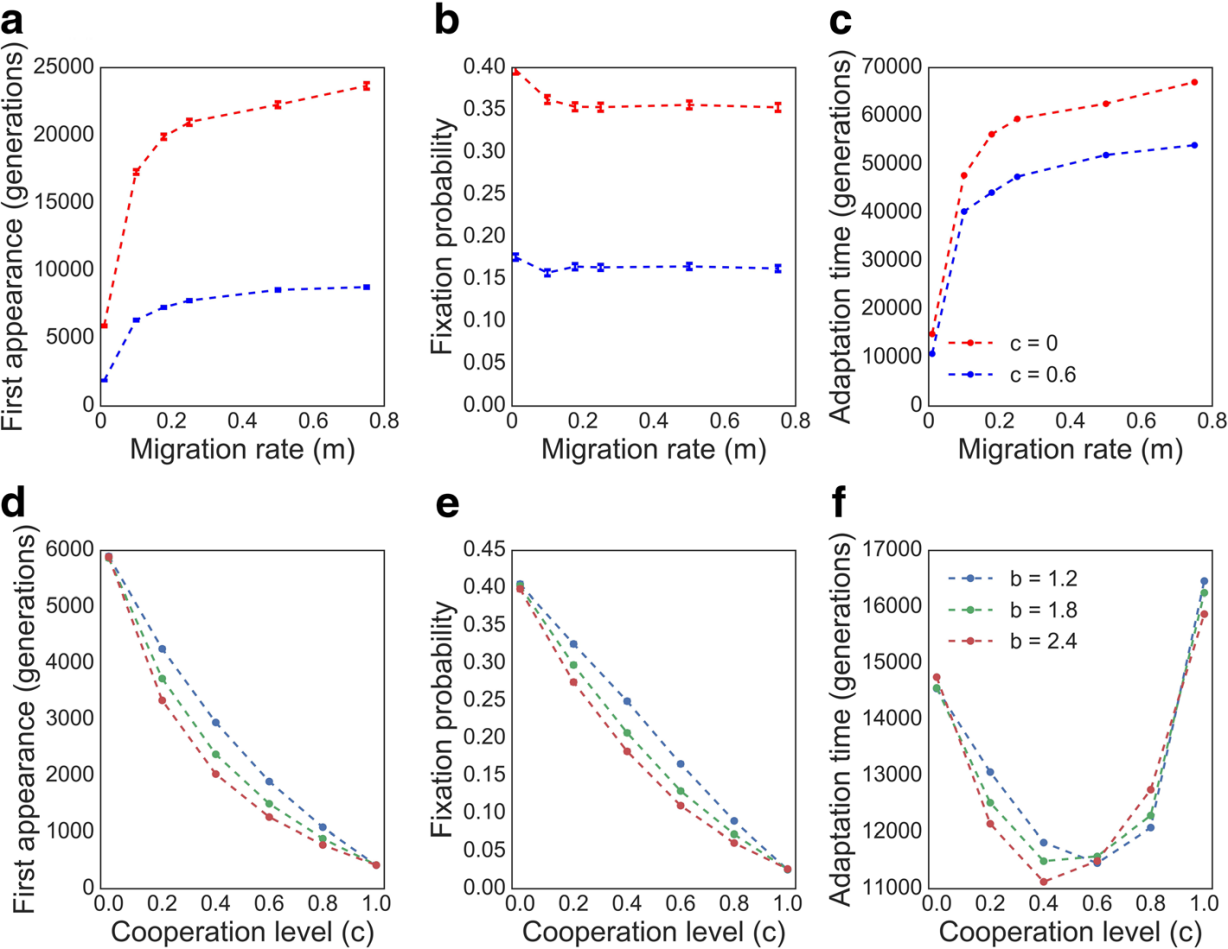
Cooperation and migration affect complex adaptation in subdivided populations. (**a**, **d**) The waiting time for the first appearance of a double mutant; (**b**, **e**) Fixation probability of a double mutant after appearance; and (**c**, **f**) Total adaptation time are plotted as functions of migration rate and cooperation levels. Dots show results averaged over ≥10,000 simulations for panels **a**,**b**,**c** and ≥50,000 simulations for **d**,**e**,**f**; bars show standard error of the mean. Additional parameter values: *r* = 0.01 , *n* = 1,000 , *k* = 10 , *μ* = 10^−5^ , *s* = 0.05 , *H* = 5. (a, b, c) *b* = 1.2, (d, e, f) *m* = 0.01

We further investigated the effects of migration rates (*m*), cooperation level (*c*), and cooperation benefit (*b*) on the peak shift process. In Fig. 3 we break down the dynamics of the double mutant fixation to the waiting time for the appearance of a double mutant (Fig. 3a and d), the fixation probability of a double mutant (Fig. 3b and e), and the overall time to adaptation (Fig. 3c and f). We observe that increasing migration increases the time to the first appearance of the double mutant (Fig. 3a) as well as mildly decreases the probability of fixation (Fig. 3b), altogether elongating the entire peak shift process (Fig. 3c) both in cooperative and non-cooperative populations.

In congruence with our analytical approximation for a panmictic population (Additional file 1: Figure S6), we found that increasing cooperation levels, or *c* values, substantially shortens the waiting time for appearance of a double mutant (Fig. 3d), but also reduces its fixation probability (Fig. 3e). Therefore, intermediate cooperation levels can minimize the adaptation time by striking a balance between shortening the waiting time for appearance of a double mutant and decreasing its fixation probability (Fig. 3f). The benefit produced from contributed resources (*b*) has a similar effect to the one observed for panmictic populations (see Additional file 1: Figure S6 for the analytic results). Higher *b* values “smooth” the adaptive landscape and decrease the waiting time for the appearance of a double mutant (Fig. 3d). On the other hand, increasing *b* reduces the advantage of the double mutant and therefore decreases its fixation probability (Fig. 3e). Of course, when the population is non-cooperative (*c* = 0), *b* doesn’t affect the results. With full cooperation (*c* = 1), all individuals within a deme have the same fitness regardless of their genotype, thus yielding the same relative fitness for all *b* values (Eq. 1).

### Population diversity

Another interesting facet of the evolutionary process of peak shifts is the population diversity. A genetically diverse population is more robust to environmental changes that change genotypes’ fitness, and is thus less likely to become extinct due to such changes [30]. In order to measure the genetic diversity, we use Shannon’s Index, normalized by the number of genotypes:

$D=\frac{-\sum_i{p}_i \ln \left({p}_i\right)}{ \ln (4)}$, where *i* ∈ {*ab*, *Ab*, *aB*, *AB*}

where *p*_*i*_ is the frequency of genotype *i* in the entire population. *D* ranges from zero to one, indicating only one genotype exists or that all genotypes are found in the population in equal frequencies, respectively.

**Fig. 4:**
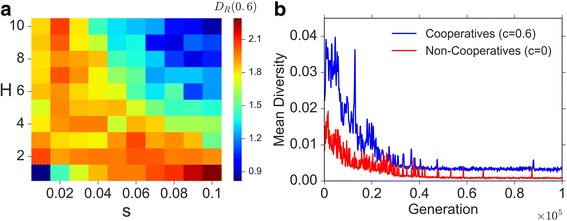
Effect of cooperation on genetic diversity during a peak shift. **a** The relative increase in diversity, *D*_*R*_(*c* = 0.6), as a function of the selection coefficient, *s*, and the double mutant coefficient, *H*. Cooperating populations are more diverse throughout the parameter range. Data is averaged over ≥200 simulations per parameter set. **b** The average genetic diversity (taken over ≥200 simulations) in a cooperative population (blue line) is higher than that of a non-cooperative population (red line) at almost every time point. Parameter values are: (**b**) *s* = 0.05 , *H* = 5; (**a**, **b**)  *n* = 1,000 , *k* = 10 , *μ* = 10^−5^ , *r* = 0.01 , *b* = 1.2 , *m* = 0.01

We define the relative increase in diversity between cooperative and non-cooperative populations to be ${D}_R(c)=\frac{D(c)- D(0)}{D(0)}$. For diversity analysis, we simulated the peak shift dynamics for 100,000 generations and recorded the diversity every 100 generations. In Fig. 4 we show the difference in diversity between cooperative and non-cooperative populations, *D*_*R*_(*c*), against varying *s* and *H* values. For all examined selection coefficients, *s* and *H*, cooperation increases the diversity relative to a non-cooperative population, usually by more than two-fold (*D*_*R*_(0.6) > 1, Fig. 4a).

During a peak shift, populations reach mutation-selection balance under two selective regimes: First before the successful double mutant appears and second after it fixates. The population diversity in the second mutation-selection balance is lower than in the first, since the selective disadvantage of the single mutant compared to the double mutant is higher than compared to the wild-type. Cooperation reduces the effective disadvantage $\left(\overset{\sim }{s}\right)$ of single mutants as well as the effective advantage of double mutants $\left(\overset{\sim }{sH}\right)$, and therefore increases diversity both before, after, and during the peak shift (Fig. 4; see also Additional file 1: SI3).

When selection is strong, cooperators achieve a peak shift faster and spend more time in the second mutation-selection balance, relative to non-cooperators (Additional file 1: SI3). Hence, strong selection diminishes the diversity advantage of cooperators (see Fig. 4a, upper-right corner). In addition, when selection is very weak, (Fig. 4a, bottom-left corner), many of the cooperating populations do not accomplish a peak shift within the time frame, and the effect of cooperation on diversity diminishes as well.

Overall, we see that a cooperative population retains, on average, higher diversity than a non-cooperative population during the entire peak shift process (Fig. 4b; see also Additional file 1: SI3). We also observe that the marked increase in diversity in cooperative populations usually involves an increase in the fixation time, due to reduction in the effective selective advantage [18, 33–35] (see Additional file 1: Figure S12). However, the fixation time is very short relative to the total peak shift time under our parameters (see Additional file 1: SI1a, SI4 and Figure S5), and only weakly affects *ρ*.

## Discussion

In this study, we have shown that cooperation can be an important factor in the evolution of complex traits. We have found that in a *public goods* scenario with strong selection, cooperative behavior can accelerate a peak shift relative to non-cooperative behavior, and the range of selection coefficients allowing this widens for low recombination rates. However, cooperation does not widen the parameter range allowing for a peak shift in our model. The effect of cooperation on adaptation time may not be monotonic, and intermediate values of cooperation (i.e. not a full investment of one’s resources in cooperative behavior) might be optimal for achieving peak shifts.

Our work belongs to a class of solutions that do not directly alter the genotype-phenotype map, but effectively smooth the adaptive landscape, and are common as means of explaining ostensibly implausible adaptive peak shifts. Some examples for this are changes in environmental conditions causing fluctuations in the adaptive landscape [36], changes in severity of selection between two connected populations [19], or a *division-of-labor* type of interactions between individuals [37]. Importantly, a cooperative population in our model will have the same ratio of mean fitness before and after a peak shift as a non-cooperative population. Therefore cooperation, as modeled here, retains the adaptive advantage observed in a peak shift of a non-cooperative population, but may accelerate the rate of the adaptation.

We also examined how cooperation affects genetic diversity. Cooperation “smooths” the adaptive landscape by decreasing selection intensity. Therefore cooperation increases genetic diversity before, during, and after a peak shift (Fig. 4 and Additional file 1: SI3). The higher genetic diversity found in cooperative populations throughout the peak shift process can influence their survival probability. Populations might encounter new parasites, predators, or abiotic environmental changes, against which some of the intermediate genotypes might have an advantage [38]. Maintaining intermediate genotypes could in such cases be a substantial advantage of cooperation.

Cooperation is a widespread biological phenomenon, with a vast body of theoretical literature supporting its evolutionary feasibility [39–44]. We chose a *public goods* type of cooperation as it is a common form of cooperation in nature [45] and therefore relevant for evolutionary processes. Different models of cooperation might yield different results, but for scenarios under which the cooperative behavior “shelters” less fit genotypes from selection, while retaining some of the advantage of the fitter genotypes, it may help accelerate the peak shift process.

The focus of our work is the effect of cooperation on the dynamics of complex adaptation, rather than the conditions leading to the emergence of cooperative behavior. We note that our results hold for populations with low relatedness (in a panmictic population) as well as populations with high relatedness (*m* ≪ 1).

Our model assumes that migration is equal between all demes (equivalent to spatial homogeneity between the demes). This assumption can be violated if migration is fitness-associated at the individual level. In this case, less fit individuals may be inclined to migrate more often in order to improve their offspring’s genotypes [46] and cooperative populations would have lower effective migration rates than non-cooperative ones. However, we do not expect this to have a qualitative effect, because, as mentioned above, the advantage of cooperative populations holds for both low and high levels of relatedness (see Figs. 1 and 2).

Recently, Komarova has shown that peak shifts in asexual populations in a spatially heterogeneous environment can be facilitated by cooperation when genes affecting cooperation also determine the fitness and cooperators directly compete with non-cooperators [37]. However, we model the loci determining the fitness as independent of cooperation, and the results are relevant for peak shifts of genes that are not directly affected by cooperation. Furthermore, we show that this can occur for sexual organisms, and even without explicit spatial constraints (e.g. panmictic populations; Fig. 1).

Multicellular, sexually reproducing organisms are an obvious fit to our model assumptions, as long as recombination rates between the relevant loci are low and selection is not too weak. Our model can also be relevant to bacteria which can face a peak shift challenge, for example, when developing antibiotic resistance [47, 48]. Furthermore, some mutations that confer antibiotic resistance carry a fitness cost, but can be compensated by additional mutations that are beneficial in the presence of antibiotics and slightly deleterious in its absence [49]. Bacteria carrying both resistance and compensation mutations in an environment currently without antibiotics would need to cross an adaptive valley to become non-resistant and uncompensated. Although bacteria do not reproduce sexually, they can perform some horizontal gene transfer [50, 51], as befitting our model. Bacterial cooperation is indeed documented: bacteria often aggregate to produce biofilms or molecules that can be considered as *public goods* [52–54]. Our results suggest that cooperative bacteria may enjoy an additional benefit of crossing adaptive valleys faster and having increased genetic diversity. Such understanding of bacterial population dynamics might be used to devise strategies to fight antibiotic resistance, as other evolutionary processes are used for predictions of efficient treatment strategies [55–59].

## Conclusions

We suggest a possible interplay between evolutionary forces and social behavior: Cooperative behavior can hasten the appearance of complex traits with increased fitness, which in turn might play a role in maintaining cooperative populations.

## Additional file

Additional file 1: contains the panmictic population analysis (SI1), the simulation design (SI2), diversity analysis (SI3) and analysis of the standard deviation of double mutants between demes (SI4). (DOCX 2235 kb)
