# Supplementary material for: With a little help from my friends: cooperation can accelerate the rate of adaptive valley crossing
Source: BMC Evol Biol. 2017 Jun 17;17:143. doi: 10.1186/s12862-017-0983-2 (PMC5473968; doi:10.1186/s12862-017-0983-2)
Supplement: Additional file 1: — contains the panmictic population analysis (SI1), the simulation design (SI2), diversity analysis (SI3) and analysis of the standard deviation of double mutants between demes (SI4). (DOCX 2235 kb) [file 12862_2017_983_MOESM1_ESM.docx]

**Additional file**

**Supplementary Information 1 – panmictic population analysis**

In this section we provide an analysis of the case of cooperating demes in a panmictic population; i.e., where each generation individuals cooperate in demes of size $k$, undergo selection, and randomly dispersed to new demes where they mate. This scenario is equivalent to our general model when the migration parameter assumes the value $m=1-\frac{1}{n}$.

In section 1a we present an analytic approximation for the time to an adaptive shift by a cooperative population, and analyze the influence of different parameters on it. We begin by analyzing the effective fitness in a cooperative population, i.e. the fitness of individuals after considering both the genotype and the effect of cooperation (1a.1). We then estimate the appearance time and fixation probability of the double mutant (1a.2, 1a.3). Finally, we derive the total time for adaptation (1a.4). In section 1b we compare between the analytic results of section 1a and the simulations.

1a. Analytic approximation

We analyze the first appearance of a double mutant and its fixation probability under a strong selection regime, similarly to [[1](#_ENREF_1)]. Under this scenario, single mutants are found in frequencies close to the mutation-selection balance, until another mutation appears in one of the single mutants, or they mate and create the double mutant via recombination, and the double mutants increase to fixation. This is one of several possible processes leading to an adaptive peak shift (as elaborated in [[2](#_ENREF_2)]), and it requires several assumptions about the parameter values [[1](#_ENREF_1), [2](#_ENREF_2)].

Population size must be large enough for single mutants to exist in the population, but not too large so that double mutants will not commonly appear:

(SA.1)

$$\frac{s}{\mu}<N\ll\left( \frac{s}{\mu} \right)^{2}$$

Single mutants ought to be sufficiently deleterious, so that their frequency could be well approximated by the deterministic values of the mutation-selection balance:

(SA.2)

$s\gg\frac{1}{N},\mu; s>\sqrt{\frac{rH}{N}};$ $s$ is not much smaller than $\frac{r}{2}$.

Selection should be strong enough for the double mutant to overcome the decomposition of mutations caused by recombination:

(SA.3)

$$sH>\frac{r}{1-r}$$

The mutation rate should be low relative to the population size, so the total adaptation time would be dominated by the waiting time for the first successful double mutant:

(SA.4)

$$N\mu\ll1$$

All the assumptions above, and other minor assumptions, are mentioned in their context of the analysis at the segments below.

1a.1. Effective selection coefficients in a cooperating population

Differently from the model presented in [[1](#_ENREF_1)], we define the fitness of each individual not only by its genotype, but also by the cooperation factors $\left( c, b \right)$, by the number of individuals in the deme ($k$), and by the fitness of the rest of the deme members. Equation 1 in the main text presents the fitness function of each individual, according to the above-mentioned factors. Hence, in order to use the methods from [[1](#_ENREF_1)], we must first define and calculate the effective fitness of each genotype, i.e. the fitness after considering both the genotype and the effect of cooperation. Since fitness is affected by the composition of the deme an individual inhabits, there are no single values representing the fitness of single mutant or the fitness of the wild type. In order to overcome this issue, we look for the mean fitness of each genotype, averaging over all possible deme compositions. We define $\tilde{s}$ and $\tilde{sH}$ as the effective selection coefficients, i.e. the average disadvantage of the single mutants and the average advantage of the double mutants relative to the wild type, after cooperation has taken place. We also mark by $\bar{\omega}_{ab,k}$ , $\bar{\omega}_{SM,k}$ and $\bar{\omega}_{AB,k}$ the mean fitness of wild type, single mutant and double mutant individuals, respectively, after considering cooperation in demes of size $k$. This allows us to formally define the effective selection coefficients, $\tilde{s}$ and $\tilde{sH}$ as follows:

S1. $\tilde{s}:=1-\frac{\bar{\omega}_{SM,k}}{\bar{\omega}_{ab,k}}$

S2. $\tilde{sH}:= \frac{\bar{\omega}_{AB,k}}{\bar{\omega}_{ab,k}}-1$

We now turn to finding the mean fitness of each genotype. In order to calculate an individual's fitness, we must know the composition of the deme it is inhabiting, and specifically, how many single mutants are found in the deme. Following Crow and Kimura 1970 [[3](#_ENREF_3)] we know that when $\frac{1}{N}\ll s$and $\mu\ll s$ and when the frequency of the single mutants is low, the population reaches a mutation-selection balance (MSB) in which the expected frequency of each of the single mutants is $\frac{\mu}{s}$. In the next analysis we will mark the expected frequency of the single mutants by $\frac{2\mu}{\tilde{s}}$, and explicitly derive $\tilde{s}$ later on. We use the MSB to approximate the frequency of the single mutants in each generation. For this approximation we need the previous assumptions of (SA.2) [[1](#_ENREF_1), [2](#_ENREF_2)].

In the current analysis we focus on a panmictic population. Until the first appearance of a double mutant, the number of single mutant $\left( SM \right)$ deme members co-inhabiting a deme of size $k$, with an individual of genotype $g$, approximately follows a binomial distribution: $X_{SM,k}\sim Bin\left( k-1,\frac{2\mu}{\tilde{s}} \right)$, where $\frac{2\mu}{\tilde{s}}$ is the frequency of $sm$ individuals in the entire population, following the mutation-selection balance frequencies.

We denote by $\omega_{g}$ the basic fitness of each individual as defined in the main text, before considering effects of cooperation $(\omega_{ab}=1, \omega_{SM}=1-s, \omega_{AB}=1+sH)$. We can now define the fitness of each genotype in a deme of size $k$ as a random variable, which depends on the number of single mutants in the deme, $X_{sm,k}$:

S3. $\omega_{g,k}:=\omega_{g}\left( 1-c \right)+\frac{\omega_{g}+X_{SM,k}(1-s)+\left( k-1-X_{SM,k} \right)}{k}cb$

By calculating the expected value of $\omega_{g,k}$ we can approximate the mean fitness of each individual in the population.

Using the linearity properties of the expectation function we get:

S4. $\bar{\omega}_{g,k}:=E\left[ \omega_{g,k} \right]=\omega_{g}\left( 1-c \right)+cb+\frac{\omega_{g}-1-E\left[ X_{SM,k} \right]s}{k}cb$

where $E\left[ X_{SM,k} \right]=\frac{2\mu}{\tilde{s}}\left( k-1 \right)$, following the expected value of the binomial distribution. Hence we can derive the mean fitness of each genotype:

S5. $\bar{\omega}_{ab,k}=1-c+cb-\frac{k-1}{k}\cdot\frac{2\mu}{\tilde{s}}scb$

S6. $\bar{\omega}_{SM,k}=\left( 1-s \right)\left( 1-c \right)+cb-\frac{s\left( 1+\frac{2\mu}{\tilde{s}}\left( k-1 \right) \right)}{k}cb$

S7. $\bar{\omega}_{AB,k}=\left( 1+sH \right)\left( 1-c \right)+cb+\frac{sH-\left( \frac{2\mu}{\tilde{s}}\left( k-1 \right) \right)s}{k}cb$

The double mutant’s effective fitness presented in S7 is accurate when the double mutants are still rare. Since the first generations after the double mutant’s first appears are the most critical in the double mutant’s fixation process, this effective fitness coefficient will serve as an approximation for the double mutant fitness throughout the process [[1](#_ENREF_1)].

By using equations (S1-2 and S5-7) we can extract $\tilde{s}$ and $\tilde{sH}$:

S8. $\tilde{s}=s\frac{1-c+\frac{cb}{k}+\frac{\left( k-1 \right)}{k}2\mu cb}{1-c+cb}$

S9. $\tilde{sH}=sH\frac{1-c+\frac{cb}{k}+\frac{\left( k-1 \right)}{k}2\mu cb}{1-c+cb}$

Substituting $s$ in S9, we can deduce that $\tilde{sH}=\tilde{s}H$, i.e. the effective selection coefficient of the double mutant equals to the effective selection coefficient of the single mutant multiplied by $H$. Notice that in the trivial case of demes of size 1 ($k=1)$, there is no cooperation, and indeed according to equations S8-9 $\tilde{s}=s$ and $\tilde{sH}=sH$.

We can further simplify $\tilde{s}$ by approximations. Note that $1-c+cb\geq1$ for any $0\leq c\leq1, b\geq1$, and that $1-c+\frac{cb}{k}\gg\frac{\left( k-1 \right)}{k}2\mu cb$ whenever $2\mu\left( k-1 \right)\ll1$. Therefore, we find that when cooperating groups are not to large compared with the mutation rate, i.e. $k\ll\frac{1}{2\mu}$, $\tilde{s}$ can be approximated by:
 S10. $\tilde{s}\approx s\frac{1-c+\frac{cb}{k}}{1-c+cb}$

Similarly, we find that:

S11. $\tilde{sH}\approx sH\frac{1-c+\frac{cb}{k}}{1-c+cb}$

We observe that $\frac{1}{k}\leq\frac{1-c+\frac{cb}{k}}{1-c+cb}\leq1$ for any $1\geq c\geq0, b\geq1$, and thus $\tilde{s}$ satisfies:

S12. $\frac{1}{k}s\leq\tilde{s}\leq s$

Interestingly, the lowest values attainable for $\tilde{s}$ decrease as the inverse of the deme size, $k$. Furthermore, selection against the single mutant in a cooperative population is linearly dependent on the original selection coefficient, $s$.

Taking the derivative of $\frac{1-c+\frac{cb}{k}}{1-c+cb}$ with respect to $c$ yields:

S13. $\frac{\partial}{\partial c}\left( \frac{1-c+\frac{cb}{k}}{1-c+cb} \right)=-\frac{k-1}{k}\frac{b}{\left( \left( b-1 \right)c+1 \right)^{2}}$

which is always negative for $1\geq c\geq0, b\geq1, k>1$, implying that $\tilde{s}$ is monotonically decreasing in $c$. Furthermore, the derivative with respect to $k$ yields:

S14. $\frac{\partial}{\partial k}\left( \frac{1-c+\frac{cb}{k}}{1-c+cb} \right)=-\frac{bc}{k^{2}\left( \left( b-1 \right)c+1 \right)}$

which is again negative for $1\geq c\geq0, b\geq1, k>1$, implying that $\tilde{s}$ is monotonically decreasing in $k$ as well, which is intuitive as increasing $k$ diminishes the relative fitness contribution of a single mutant, effectively flattening the landscape.

We also notice that changing the cooperation benefit, $b$, will change the dependency of $\tilde{s}$ in $c$ from a linear decrease for $b=1$, to monotonically decreasing convex function with an increasing curvature for higher $b$ values. This dependency will also change at the limits of possible cooperation levels from $\tilde{s}=s$ when $c=0$, to $\tilde{s}=\frac{s}{k}$ when $c=1$. This is exemplified in Fig. S1. Following S10-11, we know that varying $s, b, k$ and $c$ affects the double mutant's effective advantage ($\tilde{sH})$ exactly as it affects the single mutant's effective disadvantage $\left( \tilde{s} \right)$. Since the effective fitness of the double mutant is given by $1\boldsymbol{+}\tilde{s}H$, as opposed to the single mutant fitness: $1\boldsymbol{-}\tilde{s}$, we get that $s , b, k$ and $c$ have the opposite effect on the double mutant fitness than on the single mutant fitness.


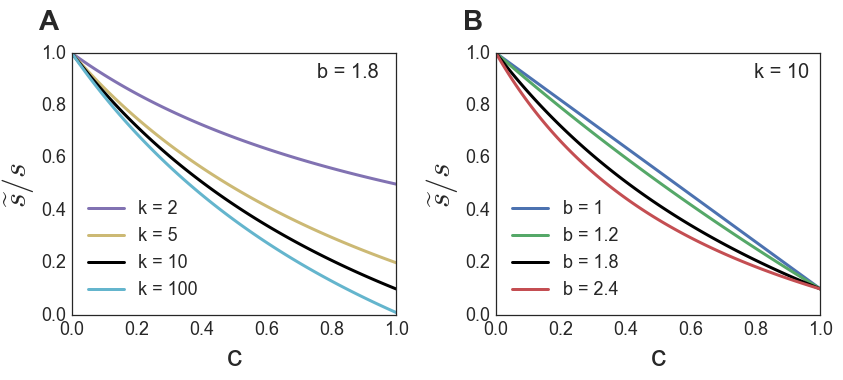


**Figure S1. Cooperation reduces the single mutant's disadvantage and the double mutant's advantage equally.** From equations S10-11 we get that $\frac{\tilde{s}}{s}=\frac{\tilde{sH}}{sH}\approx\frac{1-c+\frac{1}{k}cb}{1-c+cb}$. **(A)** By deriving this term, and setting $b=1.8$, we see that as the level of cooperation $(c)$ increases, the single mutants' disadvantage, $\tilde{s}$, decreases from $s$ (when $c=0$), to $\frac{1}{k}s$ (when $c=1$)**.** Similarly, as the level of cooperation $(c)$ increases, the double mutants' advantage, $\tilde{sH}$, decreases from $sH$ (when $c=0$), to $\frac{1}{k}sH$ (when $c=1$). **(B)** We set $k=10$ and can see that the decrease in selection coefficients is linear in $c$ for $b=1$, and convex for $b>1$. Note that the black curves represent the same parameter values in both panels ($k=10, b=1.8$), for easy comparison.

1a.2. Estimating the waiting time for the appearance of a first double mutant

Assuming the single mutant frequencies follow the approximation at mutation-selection balance, $\frac{\mu}{\tilde{s}}$ (following the constraints imposed by SA.1-2), and neglecting terms of the order of $\mu^{3}$, we find that during mutation-selection balance a fraction $r\left( \frac{\mu}{\tilde{s}} \right)^{2}$of the individuals in each generation are born double mutants as a result of recombination between $Ab$ and $aB$, a fraction $2\left( \frac{\mu}{\tilde{s}} \right)\mu$ of the individuals are born double mutants as a result of a single mutant acquiring a second mutation, and a fraction $\mu^{2}$ of the individuals are born double mutants as a result of a wild type acquiring both mutations. Thus, we can approximate $q$, the expected frequency of $AB$ offspring at a certain generation, given no $AB$ individuals exist in the previous generation [[1](#_ENREF_1)]:

S15. $q=\left( \frac{\mu}{\tilde{s}} \right)^{2}\cdot(r+2\tilde{s}+\tilde{s}^{2})$

Hence, the probability that the first $AB$ individual would appear in the population at a certain generation, given that it had not appeared earlier is:

$$1-\left( 1-q \right)^{N}$$

If random encounters of single mutants are rare enough in the population $\left( \left( \frac{\mu}{\tilde{s}} \right)^{2}\ll\frac{1}{N} \right)$ , this term can be approximated by $qN$.

Since we assume that the population genotype frequencies are approximately at the values expected from the mutation-selection balance at every generation before the double mutant appears (under the constrains of SA.1-2), the time for appearance of the first $AB$ individual $\left( T_{first} \right)$ is geometrically distributed. We can now conclude that the expected time for appearance of the first $AB$ individual is [[1](#_ENREF_1)]:

S16. $E\left[ T_{first} \right]\approx\frac{1}{qN}$

If $\tilde{s}\ll1$, we can further approximate $q$, as presented in S15, and neglect the proportion of double mutants offspring born to wild type parents:

S17. $q\approx\frac{\mu^{2}}{\tilde{s}}\left( \frac{r}{\tilde{s}}+2 \right)$

and the expected waiting time for the appearance of the first $AB$ can be approximated by:

S18. $E\left[ T_{first} \right]\approx\frac{\tilde{s}}{N\mu^{2}\left( \frac{r}{\tilde{s}}+2 \right)}$

S18 implies that $E\left[ T_{first} \right]$ is approximately linearly increasing in $\tilde{s}$ if $r\ll\tilde{s}$. If $r$ is closer to $\tilde{s}$, the relation is no longer linear, but remains monotonically increasing. Therefore, from equations S10, S13 and S18, we have that when $r$ is small relative to $\tilde{s}$, $E\left[ T_{first} \right]$ decreases approximately linearly with $c$, for $b=1$; whereas higher $b$ values change $E\left[ T_{first} \right]$ to a monotonically increasing convex function of $c$ with an increasing curvature (Fig. S2). When $r$ is larger than $\tilde{s}$, $E\left[ T_{first} \right]$ will remain monotonically increasing in $c$, with the same dependence in $b$, but further increasing $r$ values will notably decrease the slope of this relation (Fig. S2). Increases in $k$ will monotonically decrease $E\left[ T_{first} \right]$, as we observed in S14, since they will decrease the relative disadvantage of the single mutant.


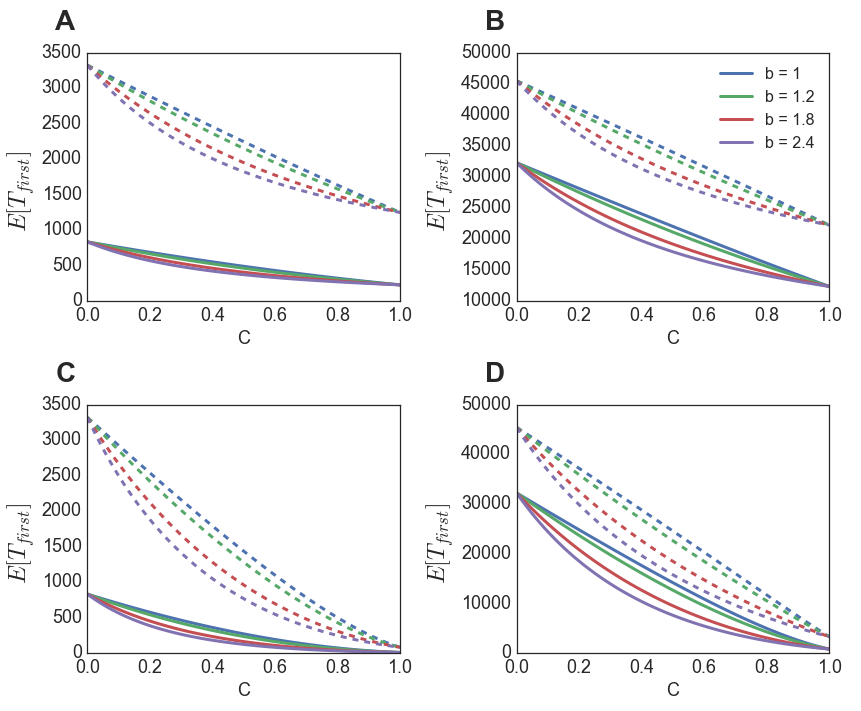


**Figure S2. Cooperation reduces the expected waiting time for the first appearance of a double mutant.** Using equations S15-16 we plot the expected number of generations until the first appearance of a double mutant as function of the cooperation level $(c)$ for $s=0.01$ (A, C) and $s=0.1$ (B, D), for deme sizes of $k=2$ (A,B) and $k=10$ (C,D). Also varied are $r$ (dashed curves for $r=0.01$ and full curves for $r=0.1$) and $b$ (curve colors) values. We can see that for all $b,s,r$ values, the expected time reduces as $c$ increases. $\mu={10}^{-5}.$

1a.3. Estimating the fixation probability of a double mutant

In order to derive the fixation probability of a double mutant we calculate the expected number of double mutant offspring born to a double mutant individual. We do so by analyzing the fitness of a double mutant relative to the population mean fitness, and by accounting for the different mating and recombination values. We assume that the recombination rate, $r$, is small enough so that $\tilde{s}H>\frac{r}{1-r}$ (SA.3). If this condition does not hold, a peak shift is expected to occur by the single mutants drifting to frequency from which they are likely to produce enough double mutants so that recombination will not break the beneficial mutation combination as they fixate – a process which is likely to be extremely long in large populations [[1](#_ENREF_1), [2](#_ENREF_2)]. We analyzed the fixation probability of the double mutant, required for derivation of the probability of a peak shift, using branching processes [[4](#_ENREF_4)].

Once a double mutant appears in the population, its fitness is $1+\tilde{s}H$, as described above (S2, S11). We approximate the population average fitness, marked by $\bar{\omega}$, at the generations following the appearance of a double mutant, by neglecting terms of the order of $\mu^{2}$:

S19. $\bar{\omega}\approx1-2\mu$

In the case of random mating, as we assume occurs, the double mutant mates with a single mutant or a wild type with probabilities $2\left( \frac{\mu}{\tilde{s}} \right)$ and $\left( 1-2\left( \frac{\mu}{\tilde{s}} \right) \right)$, respectively. In the latter case, only a proportion of $(1-r)$ of its offspring will be born double mutants. Hence, the expected number of $AB$ offspring born to an $AB$ parent, denoted by $\lambda$, is [[1](#_ENREF_1)]:

S20. $\lambda=\frac{1+\tilde{s}H}{1-2\mu}\cdot\left( \left( 1-r \right)\cdot\left( 1-2\cdot\left( \frac{\mu}{\tilde{s}} \right) \right)+2\cdot\left( \frac{\mu}{\tilde{s}} \right) \right)$

If $\mu\ll\tilde{s}$ (maintained when $\mu\ll\frac{s}{k}$ since as shown above $\frac{s}{k}\leq\tilde{s}$) then λ can be well approximated by:

S21. $\lambda\approx\left( 1-r \right)\left( 1+\tilde{s}H \right)$

Note that the expected number of $AB$ offspring, $\left( 1-r \right)\left( 1+\tilde{s}H \right)$, is greater than one if:

S22. $sH\cdot\frac{1-c+\frac{1}{k}cb}{1-c+cb}>\frac{r}{1-r}$

Therefore, if we assume that the number of offspring is Poisson distributed, we can calculate $\epsilon$, the probability that a double mutant's lineage will be driven to extinction:

$$\epsilon=\sum_{i=0}^{\infty} p\left( having i offspring \right)\cdot\epsilon^{i}=\sum_{i=0}^{\infty} \frac{e^{-\lambda}\lambda^{i}}{i!}\cdot\epsilon^{i}$$

This expression can be reformulated as follows:

$$\epsilon=\frac{e^{-\lambda}}{e^{-\lambda\epsilon}} \overset{1}{\overbrace{\sum_{i=0}^{\infty} \frac{e^{-\lambda\epsilon}\left( \lambda\epsilon\right)^{i}}{i!}}}=\frac{e^{-\lambda}}{e^{-\lambda\epsilon}}=e^{-\lambda\left( 1-\epsilon\right)}$$

We now define $\pi=1-\epsilon$ to be that fixation probability of a double mutant and we find that:

S23. $\pi=1-e^{-\lambda\pi}$

For $\lambda>1$ and $0<\pi<1$ this equation has a unique real solution:

S24. $\pi=1+\frac{1}{\lambda}W\left( -\lambda e^{-\lambda} \right)$

where $W$ is the Lambert $W$ function, also known as the product log function. Equations S20 and S24 were used to produce Fig. 1 in the main text.

If $\lambda$ is slightly above one, than $\pi$ can be well approximated by [[1](#_ENREF_1), [5](#_ENREF_5)]:

S25. $\pi\approx\frac{2\cdot\left( \lambda-1 \right)}{\lambda}$

To gain intuition for the effect various parameters have on the fixation probability, we can use S21 and S25:

$$\pi\approx\frac{2\left( \left( 1-r \right)\left( 1+\tilde{s}H \right)-1 \right)}{\left( 1-r \right)\left( 1+\tilde{s}H \right)}=2\left( \frac{\tilde{s}H-\frac{r}{\left( 1-r \right)}}{\left( 1+\tilde{s}H \right)} \right)$$

When $\tilde{s}H\ll1$ we can conclude that:

S26. $\pi\approx2\left( \tilde{s}H-\frac{r}{\left( 1-r \right)} \right)$

Using equation S11 and S26 we get that for $\lambda$ values close to 1, $\pi$ decreases with $c$ approximately linearly when $b=1$ and is a monotonic, convex function of $c$ with an increasing curvature for increasing $b$ values, analogously to the results on $E\left[ T_{first} \right]$. We note that increasing $r$ should primarily act as changing the intercept of the approximately linear relation between $\pi$ and $\tilde{s}H$, and similarly affect the relation between $\pi$ and $c$. This is shown in Fig. S3B, D, by plotting S25. We also plot equation S24 without the approximation for low $\lambda$ values (Fig. S3A, C), and see that the exact and the approximate solutions are in close agreement.


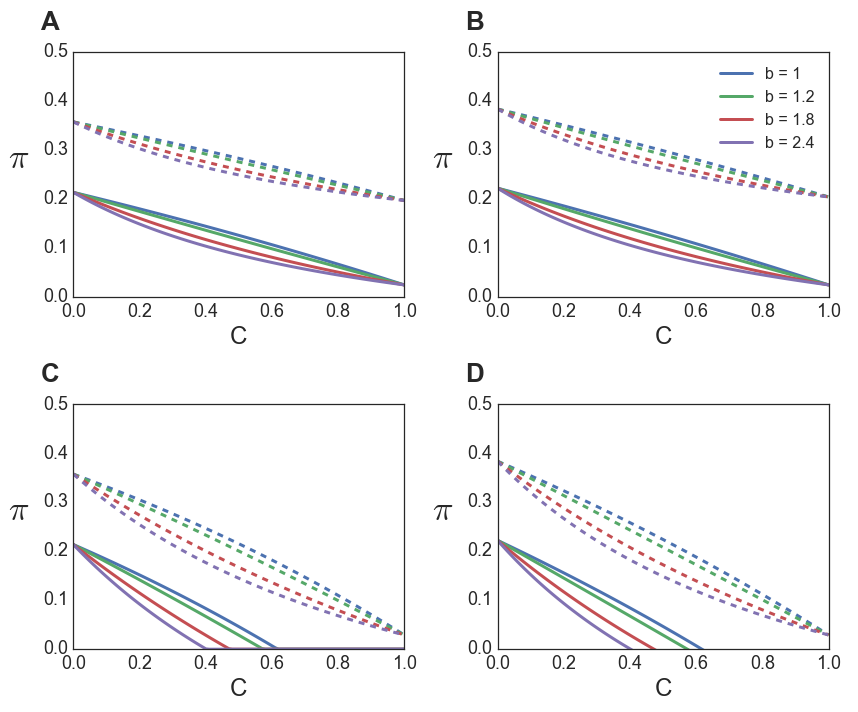


**Figure S3. Cooperation reduces the fixation probability of a lineage of a double mutant.** We plot the fixation probability of an emerging double mutant as function of the cooperation level $\left( c \right)$ according to the exact solution shown in S24 (A, C), and based on the approximation shown in S25 (B, D). We do so for several $k$ values ($k=2$ for A, B; $k=10$ for C, D), $r$ values ($r=0.01$for dashed lines and $r=0.1$ for full lines) and $b$ values (curve colors). For all $k,b,r$ values, the fixation probability decreases as $c$ increases. A fairly good agreement between the approximation and exact solution can be seen for $r=0.1$, whereas for $r=0.01$ the approximation is less accurate. When both $k$ and $c$ values are high our approximation is not valid and a peak shift will not occur in the manner describe in the main text (see curves dropping below zero at C and D). Selection coefficients used in the plots are $s=0.05,H=5$.

Finally, we examine the effect of deme size $(k)$ on the double mutant’s first appearance and fixation probability. Based on equations S16 and S24 we plot the expected waiting time for the first appearance of a double mutant and its fixation probability as a function of deme size $k$, for various cooperation levels, $c$ (Fig. S4). As seen in S14, increased $k$ reduces the selective advantage of the double mutant and selective disadvantage of single mutants. Therefore, both fixation probability and time to first appearance decrease as deme size increases.


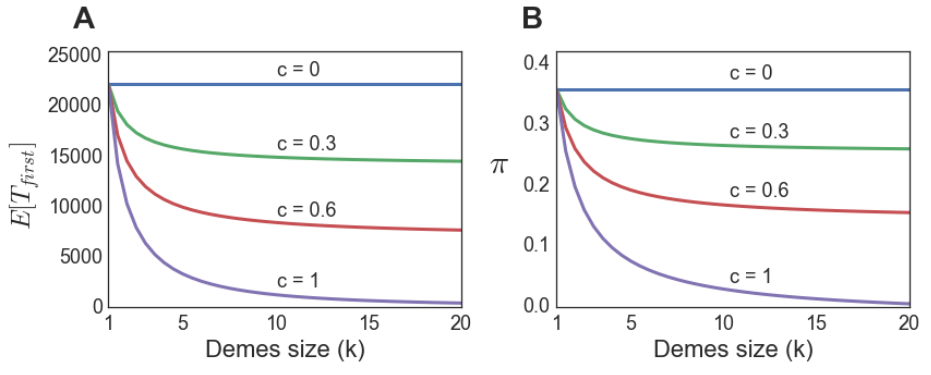


**Figure S4. First appearance of a double mutant and its fixation probability as function of deme size** $\left( \boldsymbol{k} \right)$**, in a panmictic population.** Based on equations S16 and S24, we plot the expected waiting time for the first double mutant, and its fixation probability for various cooperation levels $(c)$ and as function of the deme size $\left( k \right)$. As can be seen in S14, both the first appearance time and the fixation probability are monotonically decreasing with $k$. Parameters are: $n=10,000/k, s=0.05, H=5, \mu={10}^{-5}, r=0.01, b=1.2.$

1a.4. Estimating the total adaptation time

When the mutation rate is low relative to the population size $\left( N\mu\ll1 \right)$, the total adaptation time is dominated by the waiting time for the first successful double mutant [[6](#_ENREF_6)], i.e. a double mutant the lineage of which will fixate in the population. Therefore, in order to estimate the total adaptation time, we estimated the waiting time for the appearance of a double mutant, and the probability that its lineage will fixate in the population. The results of the main text were replicated with the inclusion of the double mutants' take-over time and proved similar (see Fig. S5 for comparison with Fig. 2A in the main text; analysis of the simulation results of Fig. 2B-D showed an even less significant effect of the fixation time).


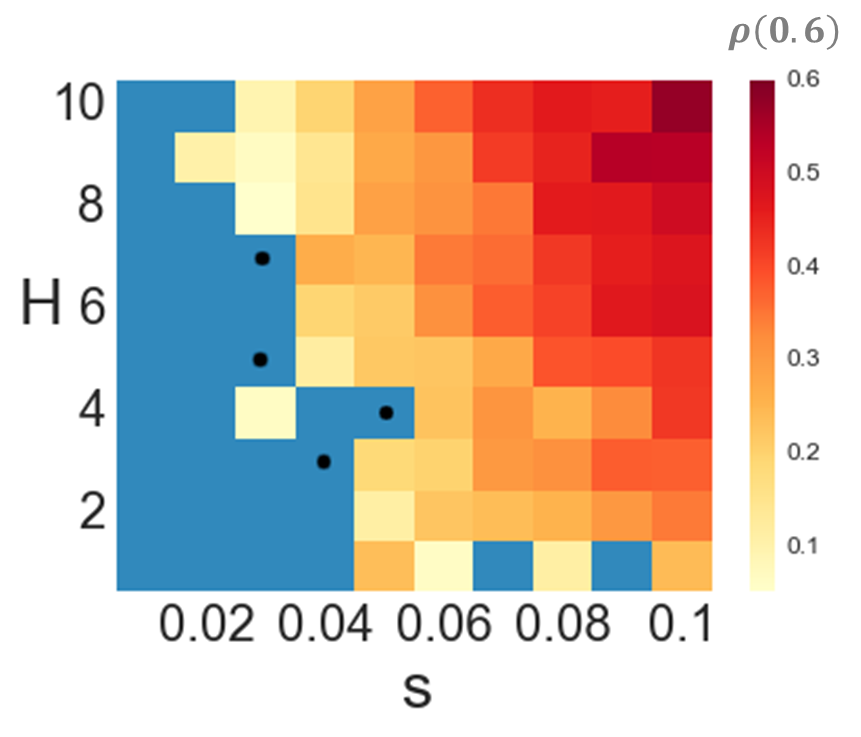


**Figure S5.** **The relative decrease in adaptation time due to cooperation, when considering the double mutants' take-over time.** This figure is analogous to Fig. 2A shown in the main text, with the addition of the take-over time of double mutants. We can see a slight decrease from the values presented in Fig. 2A since cooperation prolongs the take-over time as it decreases the double mutant's advantage. Nevertheless, since the take over time is short in comparison with the waiting time for the appearance of a successful double mutant, the decrease in $\rho\left( 0.6 \right)$ values is minor. In addition, there are four sets of $s, H$ values (marked by black dots) for which $\rho\left( 0.6 \right)$ changed from being slightly positive (in Fig. 2A) to being negative (in Fig. S5).

Combining $\pi$, the fixation probability, and $qN$, the probability of $AB$ first appearance, we can approximate the probability that a successful double mutant would appear in the next generation, given no double mutants exist in the current generation, by $\pi qN$.

Since this first appearance is geometrically distributed, we find that the expected time for appearance of an $AB$ individual that will go to fixation is:

S27. $E\left[ T_{tot} \right]\approx\frac{E\left[ T_{first} \right]}{\pi}=\frac{1}{\pi qN}$

The effects of $c$ and $b$ on the first appearance of the double mutant, its fixation probability, and the overall adaptation time are presented in Fig. S6, whereas the effects of $s$ and$H$ on the relative adaptation time between cooperative and non-cooperative populations are presented in Fig. S7, as well as in Fig. 2 in the main text.


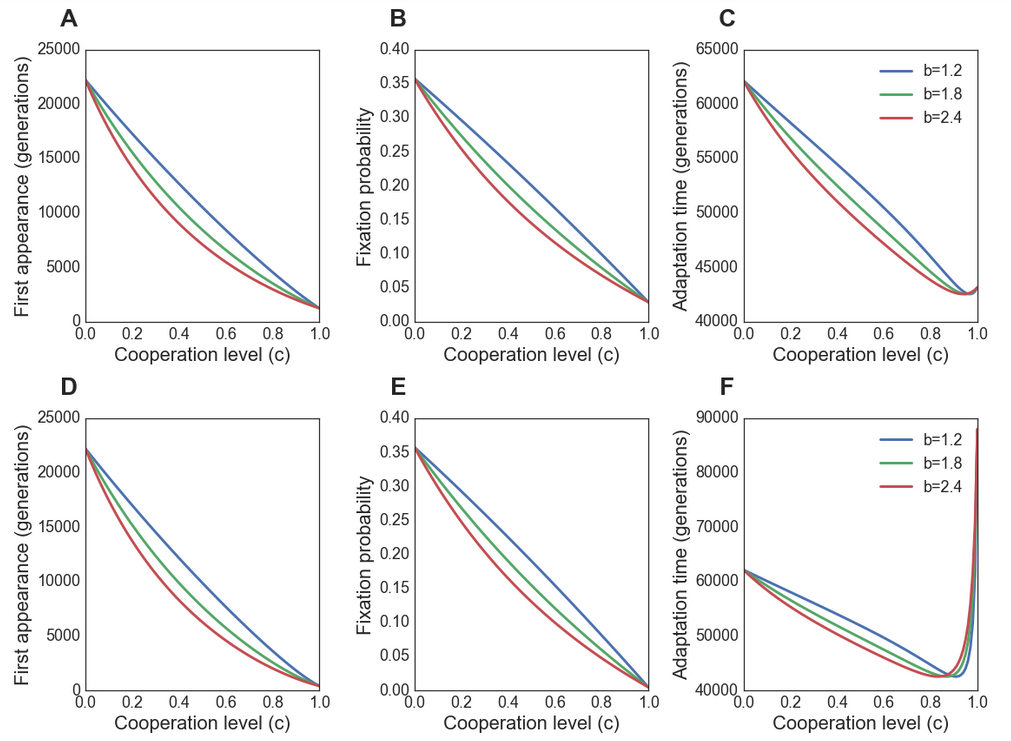


**Figure S6.** **Cooperation affects complex adaptation time in panmictic populations.** Analogously to Fig. 3 in the main text, we plot **(A,D)** the waiting time for the first appearance of a double mutant; **(B,E)** the fixation probability of a double mutant after appearance; and **(C,F)** the total adaptation time, as functions of the cooperation level. These are plotted for panmictic populations with demes of size 10 ($k=10$, panels A,B,C) and demes of size 20 ($k=20$, panels D,E,F), based on equations S15, S24 and S27. Additional parameter values: $r=0.01, n=10,000/k, \mu={10}^{-5}, s=0.05, H=5$.

In an infinite population, a double mutant lineage can fixate only if the expected number of double mutant offspring in each generation is greater than 1. In a finite yet large population, satisfying $\frac{s}{\mu}<N$, this remains a good approximation to the condition for the double mutant extinction [[1](#_ENREF_1)]. For a cooperative population we showed that the condition for this is:

S28. $\left( 1-r \right)\left( 1+sH\frac{1-c+\frac{1}{k}cb}{1-c+cb} \right)>1$

As shown earlier, $\frac{1-c+\frac{1}{k}cb}{1-c+cb}$ is a monotonically decreasing function of $c$, and therefore is $\left( 1-r \right)\left( 1+sH\frac{1-c+\frac{1}{k}cb}{1-c+cb} \right)$.

**Thus, any increase in** $\boldsymbol{c}$ **restricts the parameter range enabling** $\boldsymbol{AB}$**’s fixation.** This means that whenever a cooperative population is expected to achieve peak shift under the process we describe, a non-cooperative population $\left( c=0 \right)$ would also be expected to do so, but not necessarily vice-versa (black areas, Fig. S7 and Fig. 2 in the main text).


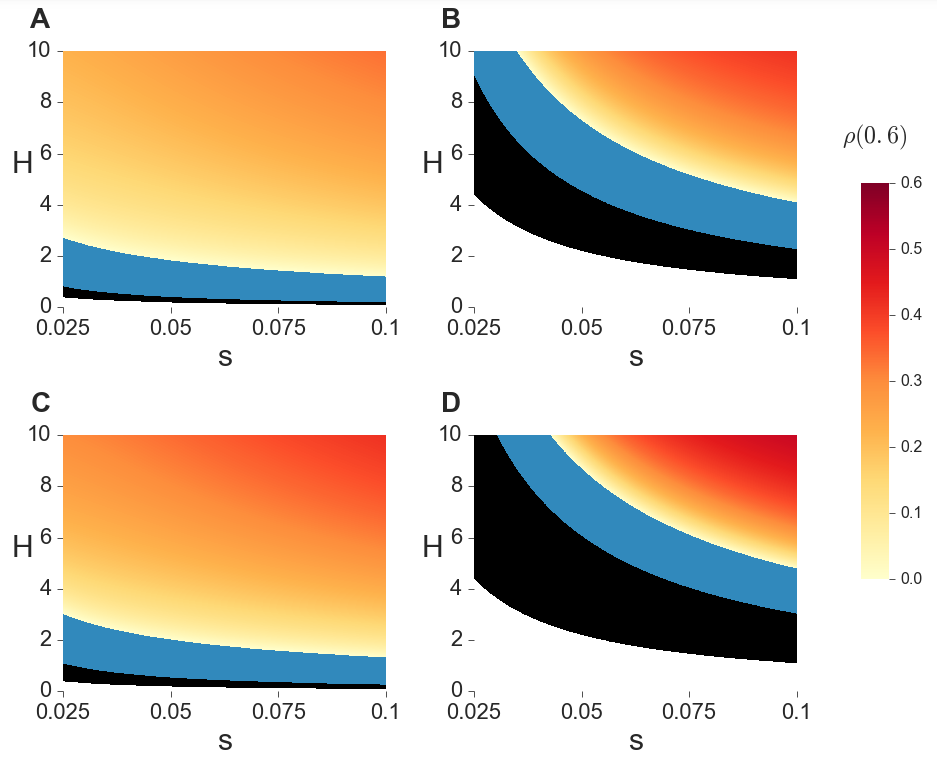


**Figure S7.** **Cooperation and selection affect adaptation time in panmictic populations.** As in Fig. 1 in the main text, we plot the relative difference in adaptation time due to cooperation ($\rho\left( 0.6 \right)$; color coding) as function of the selection coefficient, $s$ (x-axis), and the double mutant coefficient, $H$ (y-axis). The only difference from Fig. 1 in the main text is the $k$ values used: $k=5$ (panels A and B) and $k=100$ (panels C and D).

1b. Comparison between analytical approximation and simulation results

In order to verify our approximations we compare them to simulations. Fig. S8 shows the results of the simulation (blue line) and the approximation (red line), for various cooperation levels. This is shown for the time of first appearance of the double mutant (Fig. S8A, C) and the double mutant's fixation probability (Fig. S8B, D), as a function of the cooperation level ($c,$panels A-B) and as a function of demes size ($k$, panels C-D). When $c=0$, we are reduced to the results presented, in [[1](#_ENREF_1)]. We can see a good agreement between the approximation and simulation results. Note that both the waiting time for appearance of the double mutant and its fixation probability decrease with $c$ and with $k$. Further comparisons between the simulation and analytic results are presented in Fig. S9.


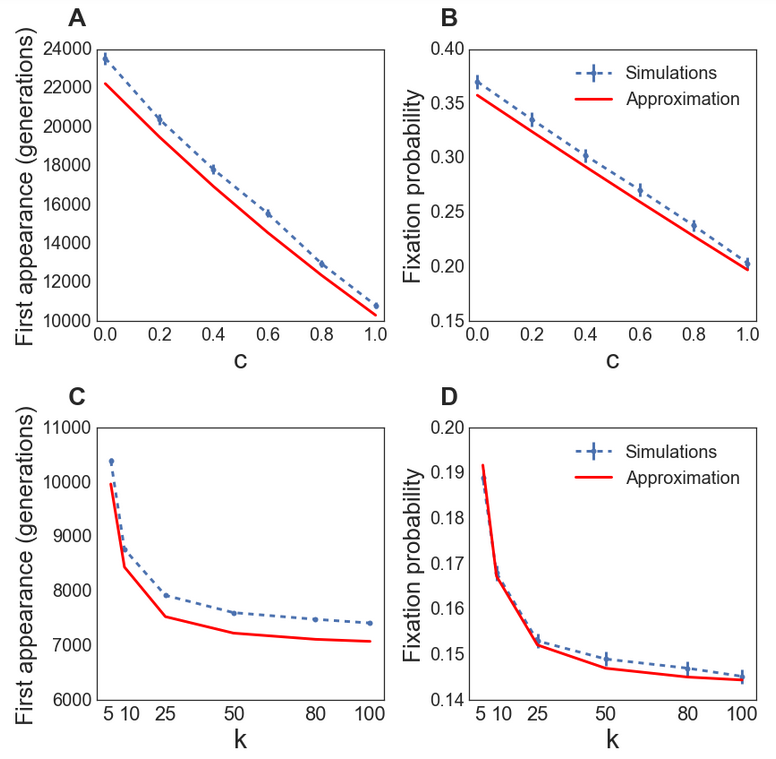


**Figure S8. Comparison between simulation and analytical approximation for various deme sizes in a panmictic population**. We plot the effect of cooperation on the first appearance of a double mutant and its fixation. Both the waiting time for appearance and the fixation probability of a double mutant decrease with the cooperation level $\left( c \right)$ (panels A-B), and with the demes size $\left( k \right)$ (panels C-D). We can see that the analytical approximation and the simulation results are in close agreement (based on at least 5,000 and 50,000 simulation runs per data point for (A,B) and (C,D), respectively. Parameters are: $s=0.05, H=5, \mu={10}^{-5}, r=0.01, b=1.2, n=10,000/k$. We use $k=2$ for panels A-B and $c=0.6$ for panels C-D.


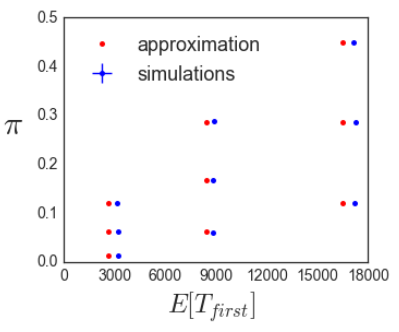


**Figure S9. Comparison between simulation and analytical approximation for various selection coefficients in a panmictic population**. Pairs of dots represent the average of simulations (blue) and the approximation results (red) for the same selection coefficients $\left( s, sH \right)$. Each column shares the same $s$ values (from left to right: $s=0.02,0.05,0.09)$ and within each column we show different $H$ values (in each column from bottom to top: $H=2,5,9$). Simulation results are based on $\sim50,000$ runs per data point. Standard errors of simulations are too small to be seen under the dots representing the average values. Other parameters are: $n=1,000, k=10, \mu={10}^{-5}, r=0.01, c=0.6, b=1.2$.

**Supplementary Information 2 – Simulation design**

The simulation is composed of a genotype frequencies vector, representing the structure and composition of the population, and of several functions, each representing a different process: selection, migration, mating and recombination, mutation and drift. One generation of the simulation ends after all functions have been applied and the population is replaced.

We initialize the simulation with a population comprised solely of wild type individuals. Every generation, we apply the selection function and calculate the fitness separately for each deme. There are $n$ demes in our model, each represented by a four-dimensional vector tracking the frequencies of the four genotypes, $G=\left\{ ab,Ab,aB,AB \right\},$ within the deme. We mark by $\omega_{g,j}$ the fitness of individuals with genotype $g$, in deme $j \left( j\in\left\{ 1,2,\ldots n \right\} \right)$, before considering the cooperation, and by $x_{g,j}$ the frequency of genotype $g$ within deme $j$. Similarly to (eq. 1) in the main text, we calculate the 'donation pool' of every deme and then derive the new fitness of each genotype, $\omega_{g,j}^{*}$ (for $g\in G$), after considering cooperation:

S29. $\omega_{g,j}^{*}=\omega_{g,j}\left( 1-c \right)+\sum_{i\in G} x_{i,j}\cdot\omega_{i,j}\cdot cb$

After that we apply the migration function to the fitness-weighted frequencies. The frequency of genotype $g\in G$ in deme $j$, after selection and migration (marked as $x_{g,j}^{'}$) is:

S30. $x_{g,j}^{'}=\left( 1-m \right)\cdot x_{g,j}\cdot\omega_{g,j}^{*}+\sum_{j\neq d=1}^{n} x_{g,d}\cdot\omega_{g,d}^{*}\cdot\frac{m}{n-1}$

After selection and migration, we apply the mating-recombination function. We calculate the frequencies of the genotypes in the next generation, within each deme, according to the frequencies in the parent generation, and the recombination rate, $r$. Finally, we apply mutation. Note that we do not normalize the results after applying the selection function, but rather after applying the migration function. Thus, demes with higher mean fitness would export more individuals (also known as “hard selection”{Christiansen, 1975 #7} [[7](#_ENREF_7)]).

The last phase in each generation is drift, simulated by drawing $k$ random numbers from a multinomial distribution based on the relative genotype frequency in each deme, and repopulating the demes. We then transform the quantities of every genotype back into frequencies. After applying all the functions, and initiating the new frequencies vector, the simulation proceeds to the next generation.

The simulations include three stages: In the first stage, a population comprised solely by wild types evolves towards a mutation-selection balance. We do so by letting each simulation run for 5,000 generations to evolve towards a mutation-selection balance. Using analysis based on [[8](#_ENREF_8)], we could estimate the time it would take a large population to reach mutation-selection balance. However, in cases where the population is divided to demes with limited migration, there is no mutation-selection balance, but rather mutation-selection-migration-drift balance. Therefore we simulated and compared the frequencies of the single mutants at two time points: after 5,000 and 10,000 generations, for more than 1,000 repetitions. We found no significant difference between the frequencies at the two time points.

At this stage we define the fitness of genotype $AB$ to be $0$ in order to reach mutation-selection balance while no double mutant is yet formed. Then, in the second stage, we update the fitness of $AB$ to be $1+sH$. We let the simulation run until a double mutant appears for the first time. This allows us to estimate the expected time for the appearance of a double mutant. In the third stage, the double mutant either becomes extinct or fixates in the population (determined by reaching a frequency of 0.99). By recording the success frequency of the double mutant over many simulations (number of double mutant fixations divided by total number of runs) we can estimate the probability that a double mutant will fixate. Combining these two measures (expected first appearance time and fixation probability) we can estimate the expected waiting time for the appearance of a double mutant that fixates (See SI1a).

The parameters we use are $n,k,r,\mu,s,H,b,c,m$, as defined in Table 1 in the main text.

1. Fitness

We mark by $\left( \begin{matrix} x_{ab,j} & x_{Ab,j} & x_{aB,j} & x_{AB,j} \end{matrix} \right)$ the frequencies of genotypes $ab,Ab,aB,AB$ respectively in deme $j$. For deme $j$ we calculate the pool of fitness donations, $pool_{j}$, using matrix product:

S31. $pool_{j}=\left( \begin{matrix} x_{ab,j} & x_{Ab,j} & x_{aB,j} & x_{AB,j} \end{matrix} \right)\left( \begin{matrix} 1\cdot c\cdot b \\ \left( 1-s \right)\cdot c\cdot b \\ \left( 1-s \right)\cdot c\cdot b \\ \left( 1+sH \right)\cdot c\cdot b \end{matrix} \right)$

After calculating the pool we can calculate the new fitness of every individual, and by that, the frequency of every genotype after applying selection. The new frequencies of genotypes in deme $j$ would be:

S32. $\left( \begin{matrix} x_{ab,j}^{'} \\ x_{Ab,j}^{'} \\ x_{aB,j}^{'} \\ x_{AB,j}^{'} \end{matrix} \right)=\left( \begin{matrix} x_{ab,j}\cdot& \left[ 1\cdot\left( 1-c \right)+pool_{j} \right] \\ x_{Ab,j}\cdot& \left[ \left( 1-s \right)\left( 1-c \right)+pool_{j} \right] \\ x_{aB,j}\cdot& \left[ \left( 1-s \right)\left( 1-c \right)+pool_{j} \right] \\ x_{AB,j}\cdot& \left[ \left( 1+sh \right)\left( 1-c \right)+pool_{j} \right] \end{matrix} \right)$

Where the vector $\left( \begin{matrix} x_{ab,j}^{'} & x_{Ab,j}^{'} & x_{aB,j}^{'} & x_{AB,j}^{'} \end{matrix} \right)$ represents the frequencies of the genotypes after selection.

1. Migration

According to (S25), we can define a migration matrix that would simulate the change in the population composition due to migration. For instance, if there are 3 demes in the population, the matrix would be defined as follows:

$$M=\left( \begin{matrix} 1-m & 0 & 0 & 0 & m/2 & 0 & 0 & 0 & m/2 & 0 & 0 & 0 \\ 0 & 1-m & 0 & 0 & 0 & m/2 & 0 & 0 & 0 & m/2 & 0 & 0 \\ 0 & 0 & 1-m & 0 & 0 & 0 & m/2 & 0 & 0 & 0 & m/2 & 0 \\ 0 & 0 & 0 & 1-m & 0 & 0 & 0 & m/2 & 0 & 0 & 0 & m/2 \\ m/2 & 0 & 0 & 0 & 1-m & 0 & 0 & 0 & m/2 & 0 & 0 & 0 \\ 0 & m/2 & 0 & 0 & 0 & 1-m & 0 & 0 & 0 & m/2 & 0 & 0 \\ 0 & 0 & m/2 & 0 & 0 & 0 & 1-m & 0 & 0 & 0 & m/2 & 0 \\ 0 & 0 & 0 & m/2 & 0 & 0 & 0 & 1-m & 0 & 0 & 0 & m/2 \\ m/2 & 0 & 0 & 0 & m/2 & 0 & 0 & 0 & 1-m & 0 & 0 & 0 \\ 0 & m/2 & 0 & 0 & 0 & m/2 & 0 & 0 & 0 & 1-m & 0 & 0 \\ 0 & 0 & m/2 & 0 & 0 & 0 & m/2 & 0 & 0 & 0 & 1-m & 0 \\ 0 & 0 & 0 & m/2 & 0 & 0 & 0 & m/2 & 0 & 0 & 0 & 1-m \end{matrix} \right)$$

This means that an individual from deme 1 remains in its deme with probability $\left( 1-m \right)$, migrates to deme 2 with probability $\frac{m}{2}$ , or migrates to deme 3 with probability $\frac{m}{2}$.

Therefore, for a frequencies vector $V$ as defined above (every 4 consecutive coordinates represent genotype frequencies of one deme), where each value represents genotype frequency before migration, the frequencies after the migrations, marked by $V'$, are calculated by:

$$V^{'}=M\cdot V$$

1. Mating and recombination

Let $x_{g,j}\in\{x_{ab,j}, x_{Ab,j}, x_{aB,j}, x_{AB,j}\}$ be the frequency of genotype $g$ in a deme $j$ in a specific generation, and $x_{g,j}^{'}$ the frequency of genotype $g$ in that same deme in the next generation. By this we get:

S33. $\left( \begin{matrix} x_{ab,j}^{'} \\ x_{Ab,j}^{'} \\ x_{aB,j}^{'} \\ x_{AB,j}^{'} \end{matrix} \right)=\left( \begin{matrix} x_{ab,j}^{2}+x_{ab,j}\cdot x_{Ab,j}+x_{ab,j}\cdot x_{aB,j}+x_{ab,j}\cdot x_{AB,j}\cdot\left( 1-r \right)+x_{Ab,j}\cdot x_{aB,j}\cdot r \\ x_{Ab,j}\cdot x_{ab,j}+x_{Ab,j}^{2}+x_{Ab,j}\cdot x_{aB,j}\cdot\left( 1-r \right)+x_{Ab,j}\cdot x_{AB,j}+x_{ab,j}\cdot x_{AB,j}\cdot r \\ x_{aB,j}\cdot x_{ab,j}+x_{aB,j}\cdot x_{Ab,j}\cdot\left( 1-r \right)+x_{aB,j}^{2}+x_{aB,j}\cdot x_{AB,j}+x_{ab,j}\cdot x_{AB,j}\cdot r \\ x_{AB,j}\cdot x_{ab,j}\cdot\left( 1-r \right)+x_{AB,j}\cdot x_{Ab,j}+x_{AB,j}\cdot x_{aB,j}+x_{AB,j}^{2}+x_{Ab,j}\cdot x_{aB,j}\cdot r \end{matrix} \right)$

1. Mutation function

The frequency of each genotype after mutation takes place can be written as:

S34. $\left( \begin{matrix} x_{ab,j}^{'} \\ x_{Ab,j}^{'} \\ x_{aB,j}^{'} \\ x_{AB,j}^{'} \end{matrix} \right)= \overset{mutation matrix}{\overbrace{\left( \begin{matrix} \left( 1-\mu\right)^{2} & \mu\cdot(1-\mu) & \mu\cdot(1-\mu) & \mu^{2} \\ \mu\cdot(1-\mu) & \left( 1-\mu\right)^{2} & \mu^{2} & \mu\cdot(1-\mu) \\ \mu\cdot(1-\mu) & \mu^{2} & \left( 1-\mu\right)^{2} & \mu\cdot(1-\mu) \\ \mu^{2} & \mu\cdot(1-\mu) & \mu\cdot(1-\mu) & \left( 1-\mu\right)^{2} \end{matrix} \right)}}\left( \begin{matrix} x_{ab,j} \\ x_{Ab,j} \\ x_{aB,j} \\ x_{AB,j} \end{matrix} \right)$

In this notation, $x_{g,j}$ represent the frequency of genotype $g$ in a specific deme $j$ before mutation and $x_{g,j}^{'}$ represents the frequency of genotype $g$ in that same deme after mutation takes place. Since the mutation occurrences are independent variables, each deme is multiplied by the same matrix. Moreover, the probability of mutation occurrence is homogenous in time; therefore the same matrix is valid for the entire simulation.

Simulations were performed using Python 3.3. When multiple parameter sets were examined, assignments with random selection of the desired parameter sets were sent to a computer cluster until the pre-determined number of runs was satisfied for each parameter set. Hence the statements of 'at least ## simulations' found in the main text.

**Supplementary Information 3 – Diversity analysis**

In this section we examine how genetic diversity during a peak shift process is affected by cooperation. Since a peak shift process is composed of several stages, each of which can have a different effect on the population diversity, we chose to compare the diversity in each stage separately. We start by recording simulations that ended with a successful peak shift (limited by 100,000 generations; see Fig. S10). For each simulation we record two time points which define the beginning of each stage of the peak shift process:

- $T_{1}$ – The generation of the first appearance of a double mutant that will eventually fixate (in contrast with appearance of double mutants that appear and then become extinct).
- $T_{2}$ – The generation in which the double mutants reached a frequency of 0.99 for the first time.

These two time points divide the process to three phases:

1. MSB1 – First mutation-selection balance. The time until the first appearance of a successful double mutant.
2. Takeover – The phase between the first appearance of a successful double mutant and its fixation.
3. MSB2 – Second mutation-selection balance, after the fixation of the double mutants.

For simulation $j$ let $t_{1}^{j}, t_{2}^{j}$ be the time of the first appearance of a successful double mutant and the time of its fixation, respectively. We normalize each generation $i$ in simulation $j$ to its relative location within its phase in the following manner:

S35. $i^{'}=\left\{ \begin{matrix} \frac{i}{t_{1}^{j}}\cdot T_{1} & i\leq t_{1}^{j} \\ T_{1}+\frac{i-t_{1}^{j}}{t_{2}^{j}-t_{1}^{j}}\cdot\left( T_{2}-T_{1} \right) & t_{1}^{j}<i\leq t_{2}^{j} \\ T_{1}+T_{2}+\frac{i-t_{2}^{j}}{100000-t_{2}^{j}}\cdot\left( 100000-T_{2} \right) & t_{2}^{j}<i\leq100,000 \end{matrix} \right.$

After normalization, we smooth the diversity of the simulations with a 10 generation moving average and plot the results. In the plots presented below, we show that cooperative populations attain higher diversity during the first and the second mutation-selection balances. In addition, during the takeover the cooperative population reaches higher levels of diversity. We can see that occasionally the diversity in a non-cooperative population is higher than in a cooperative population, but this happens only for relatively short periods and mostly when the two populations are not ‘synchronized’: For example, if the cooperative population has already crossed the valley and is in the second mutation-selection balance (after fixation) while the non-cooperative population is still in the first stage (see s=0.09, H=9 plot; generations ~10,000-20,000). Nevertheless, as shown in the Fig. 4 in the main text, the average diversity in the entire process is almost always higher for cooperative populations.


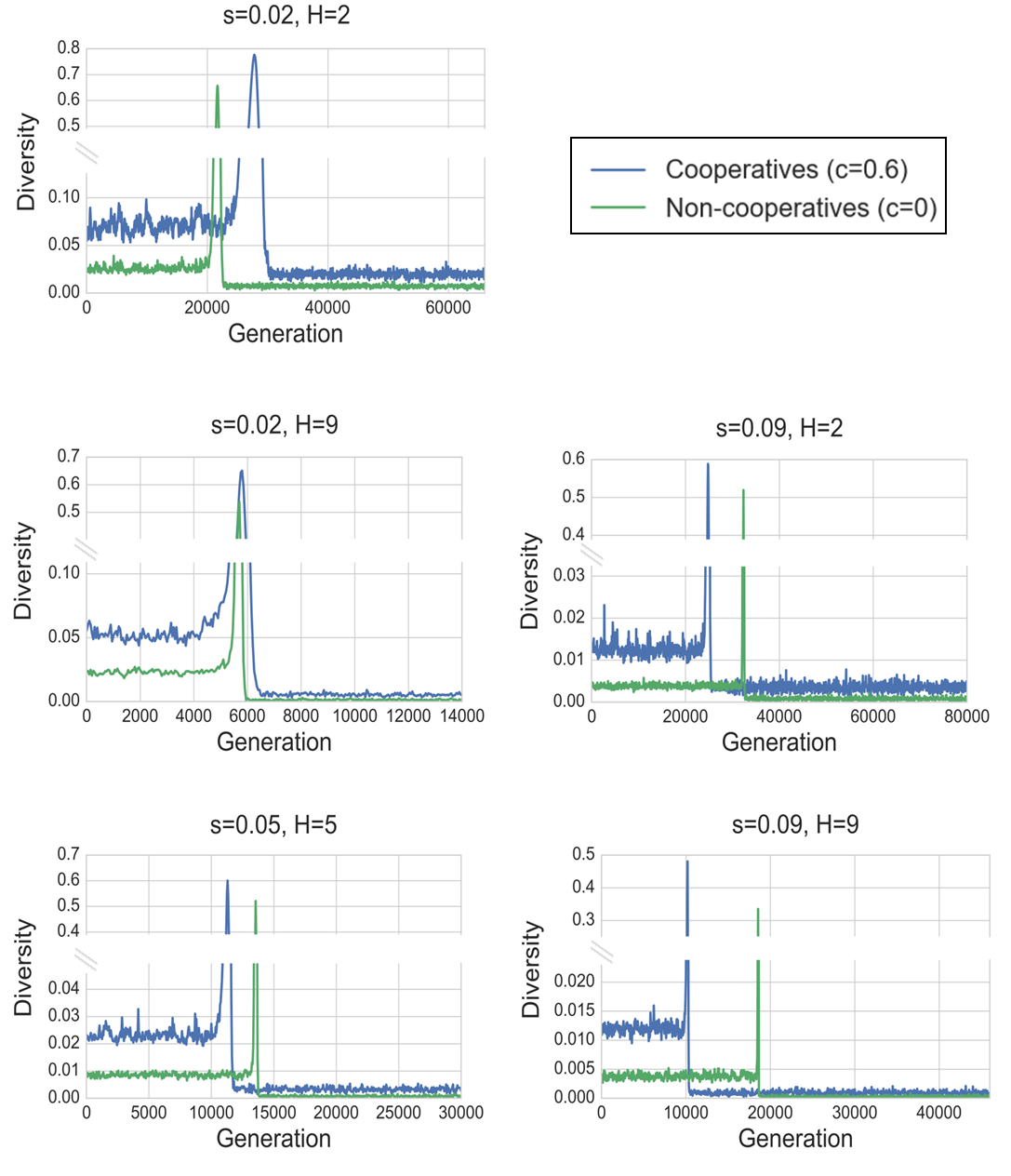


**Figure S10. Mean diversity is higher for cooperative population.** We plot the mean diversity of cooperative and non-cooperative populations, where the generations are normalized to the different phases (MSB1, Takeover, MSB2) as explained above. For each phase the diversity in a cooperative population is higher than in a non-cooperative population. Each plot is based on at least 180 simulations. Note that the y-axes are broken.

Parameters are: $n=1,000, k=10, \mu={10}^{-5}, r=0.01, c=0.6, b=1.2, m=0.01$

The relative increase in diversity due to cooperation shown in the main text (Fig. 4) is averaged over 100,000 generations. To make sure that our results are insensitive to the number of generations chosen, we examined the average diversity for different time durations, and found that the results are robust (Fig. S11).


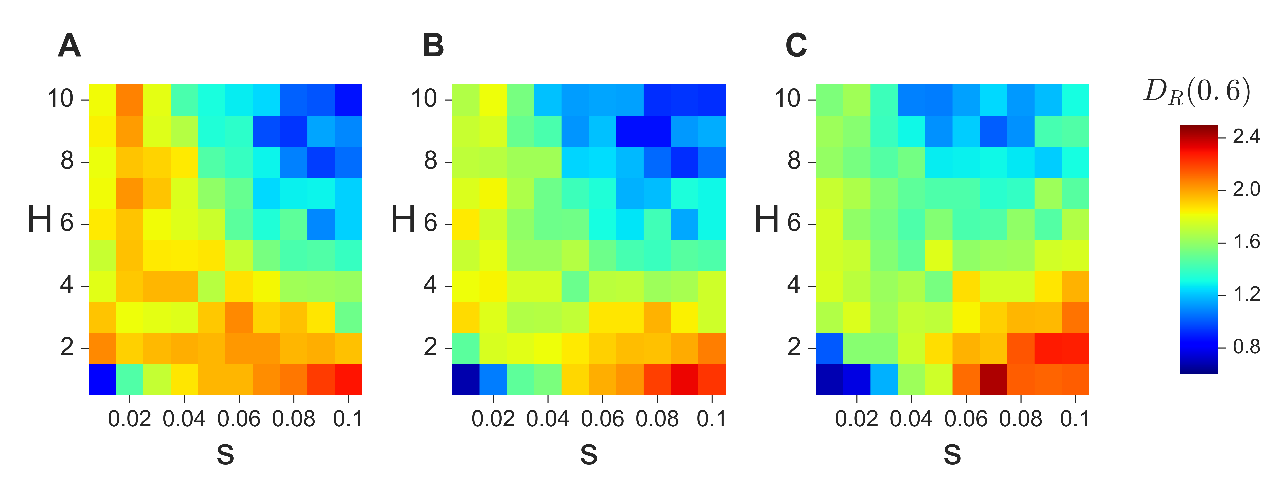


**Figure S11. Effect of cooperation on genetic diversity during a peak shift.** The relative increase in diversity, $D_{R}\left( 0.6 \right)$, is plotted as function of the selection coefficient $\left( s \right)$ and the double mutant coefficient $\left( H \right)$. All three sub-figures are based on same simulations (Data is averaged over $\geq200$ simulations per parameter set), each spanning 100,000 generations. **(A)** The average diversity was calculated over all 100,000 generations (exactly as in Fig. 4A in the main text); **(B)** over the first 50,000 generations; and **(C)** over the first 25,000 generations. All three figures show similar results, and for all $s, H$ values $D_{R}\left( 0.6 \right)>0$, i.e. cooperation increases the diversity. Parameter values are: $s=0.05, H=5, n=1,000, k=10, \mu={10}^{-5},r=0.01, c=0.6, b=1.2, m=0.01$*.*

**Supplementary Information 4 – Standard deviation of double mutants between demes**

In this section we examined the variation in the number of double mutants between demes during the fixation process. A peak shift can occur either by a rapid takeover of double mutants occupying entire demes, or by a gradual increase of the number of double mutants in each deme. The spread of double mutants in the population can be expressed by the variance of the number of double mutants between the demes:

S36. $\sigma_{AB}^{2}=\sum_{d=1}^{n} \frac{\left( AB_{d}-\bar{AB} \right)^{2}}{n}$

Where $AB_{d}$ is the number of double mutants in deme $d$, and $\bar{AB}$ is the average number of double mutants per deme $\left( \frac{\sum_{d=1}^{n} AB_{d}}{n} \right)$.

A population composed of demes with a similar number of double mutants would have low variance, whereas high variance is expected when the population is composed of demes fully inhabited by double mutants and demes completely devoid of them. We find that the cooperation level ($c$) does not influence the maximum deme variance attained during a peak shift (compare red to blue curves in Fig. S12), indicating that the highest value of variance obtained during the fixation process is similar with or without cooperation. However, the time needed for the double mutant spread in a cooperative population is both longer and more variable than in a non-cooperative one.


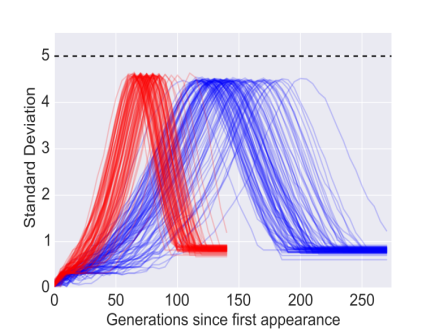

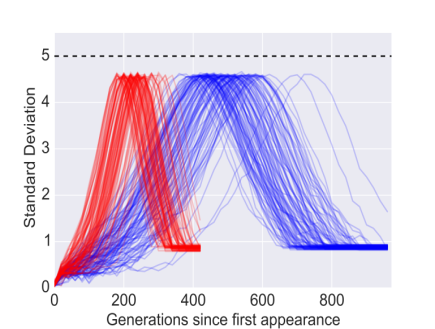


A

B


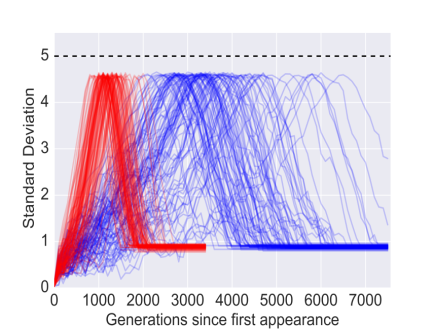

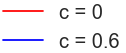


C

**Figure S12. The change in standard deviation of double mutants across demes during a peak shift.** Standard deviation of the number of double mutants between demes $\left( \sigma_{DM} \right)$ is plotted for 100 simulation runs of a cooperative population ($c=0.6$, blue) and a non-cooperative population ($c=0$, red), under several sets of selection coefficients: $s=0.1, h=10$ **(A),** $s=0.05, h=5$ **(B)** and $s=0.02, h=2$ **(C)**. Each simulation begins from the first successful double mutant (a double mutant that fixates in the population) until 99% of the population are double mutants (yielding $\sigma_{DM}<1$). The dashed line represents the theoretical boundary of the $\sigma_{DM}$ value, attained when half of the demes are inhabited solely with double mutants, and the other half is empty of double mutants. The qualitative dynamics are very similar for the different selection coefficients (compare A, B and C) though the scale is different. Other parameters: $n=1,000, k=10, \mu={10}^{-5}, r=0.01, b=1.2, m=0.01$.

**References**

1. Hadany L: **Adaptive peak shifts in a heterogenous environment**. *Theoretical Population Biology* 2003, **63**(1):41-51.

2. Weissman DB, Feldman MW, Fisher DS: **The rate of fitness-valley crossing in sexual populations**. *Genetics* 2010, **186**(4):1389-1410.

3. Crow JF, Kimura M: **An introduction to population genetics theory**. *An introduction to population genetics theory* 1970.

4. Harris TE: **Branching processes**. *The Annals of Mathematical Statistics* 1948:474-494.

5. Eshel I: **On the survival probability of a slightly advantageous mutant gene with a general distribution of progeny size—a branching process model**. *Journal of mathematical biology* 1981, **12**(3):355-362.

6. Weissman DB, Desai MM, Fisher DS, Feldman MW: **The rate at which asexual populations cross fitness valleys**. *Theoretical population biology* 2009, **75**(4):286-300.

7. Christiansen FB: **Hard and soft selection in a subdivided population**. *American Naturalist* 1975:11-16.

8. Gordo I, Dionisio F: **Nonequilibrium model for estimating parameters of deleterious mutations**. *Physical Review E* 2005, **71**(3):031907.
